# Cytomegalovirus infection lengthens the cell cycle of granule cell precursors during postnatal cerebellar development

**DOI:** 10.1172/jci.insight.175525

**Published:** 2024-06-10

**Authors:** Cathy Yea Won Sung, Mao Li, Stipan Jonjic, Veronica Sanchez, William J. Britt

**Affiliations:** 1 Department of Microbiology, University of Alabama at Birmingham, School of Medicine, Birmingham, Alabama, USA.; 2 Laboratory of Hearing Biology and Therapeutics, National Institute on Deafness and Other Communication Disorders (NIDCD), NIH, Bethesda, Maryland, USA.; 3 Department of Pediatrics, University of Alabama at Birmingham, School of Medicine, Birmingham, Alabama, USA.; 4 Department of Histology and Embryology and; 5 Center for Proteomics, Faculty of Medicine, University of Rijeka, Rijeka, Croatia.; 6 Department of Neurobiology, University of Alabama at Birmingham, School of Medicine, Birmingham, Alabama, USA.

**Keywords:** Inflammation, Neuroscience, Neurological disorders

## Abstract

Human cytomegalovirus (HCMV) infection in infants infected in utero can lead to a variety of neurodevelopmental disorders. However, mechanisms underlying altered neurodevelopment in infected infants remain poorly understood. We have previously described a murine model of congenital HCMV infection in which murine CMV (MCMV) spreads hematogenously and establishes a focal infection in all regions of the brain of newborn mice, including the cerebellum. Infection resulted in disruption of cerebellar cortical development characterized by reduced cerebellar size and foliation. This disruption was associated with altered cell cycle progression of the granule cell precursors (GCPs), which are the progenitors that give rise to granule cells (GCs), the most abundant neurons in the cerebellum. In the current study, we have demonstrated that MCMV infection leads to prolonged GCP cell cycle, premature exit from the cell cycle, and reduced numbers of GCs resulting in cerebellar hypoplasia. Treatment with TNF-α neutralizing antibody partially normalized the cell cycle alterations of GCPs and altered cerebellar morphogenesis induced by MCMV infection. Collectively, our results argue that virus-induced inflammation altered the cell cycle of GCPs resulting in a reduced numbers of GCs and cerebellar cortical hypoplasia, thus providing a potential mechanism for altered neurodevelopment in fetuses infected with HCMV.

## Abstract 2

Cytomegalovirus infection–induced CNS inflammation lengthens the cell cycle of granular cell precursors and alters cerebellar cortical development in newborn mice.

## Graphical abstract

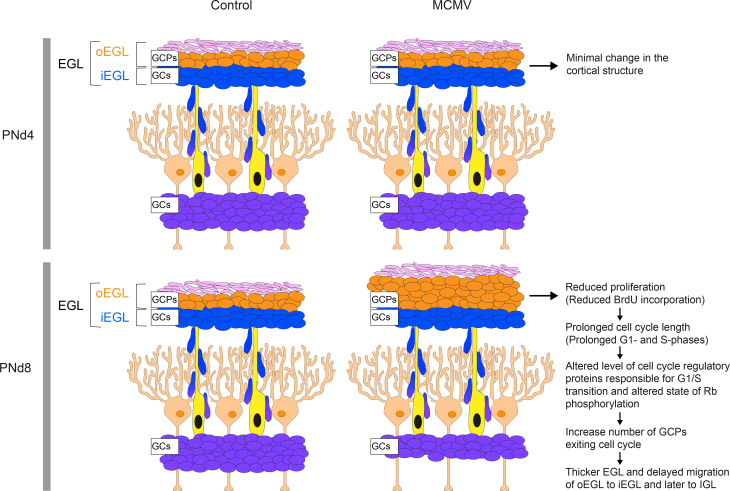

## Introduction

Congenital human cytomegalovirus (cCMV) infection is the most common intrauterine infection worldwide, affecting 0.2%–1.2% of all live births. Approximately 5%–15% of infants with cCMV (~3,000/year in the United States) will develop long-term neurodevelopmental sequelae. Neuroimaging of infants with cCMV infection has documented altered brain morphogenesis, including ventriculomegaly, microcephaly, lissencephaly, cortical dysplasia, periventricular calcifications, and cerebellar hypoplasia (1). Disordered morphogenesis of the developing brain associated with cCMV infection is almost always symmetric, a finding in contrast to infections with agents that cause necrotizing lesions in the brain, such as herpes simplex virus. Histopathological findings in the brains of infected fetuses and necropsies of newborn infants with cCMV infection have included monocytic infiltration, reactive gliosis, and foci of CD8^+^ T cell aggregates (2). Of note, histopathology is frequently observed not only in regions containing human CMV–infected (HCMV-infected) cells but also in areas of the brain without evidence of virus infection, suggesting indirect effects such as virus-induced inflammation contribute to central nervous system (CNS) damage (2). While the histopathology associated with cCMV is well described, the pathogenesis of HCMV-induced CNS damage remains undefined. We developed a mouse model of HCMV CNS infection of the developing brain in which newborn mice are infected i.p. with a nonlethal dose of murine CMV (MCMV), a virus with a genetic composition and replication program that is similar to HCMV (3). Following infection, MCMV disseminates to peripheral organs, including hematogenous spread to the CNS. Although this model does not recapitulate in utero transmission of the virus, it takes advantage of findings that have shown that the newborn mouse is neurodevelopmentally similar to a mid to late second trimester human fetus (4, 5).

The cerebellum of rodents develops postnatally, reaching maturity in mice by P21 (6, 7). Cerebellar granule cell precursors (GCPs) are the most abundant cell type in the developing cerebellum. Following a sequence of proliferation, differentiation, and migration, GCPs form the laminar structure of the cerebellar cortex, which consists of the external granule layer (EGL), molecular layer (ML), Purkinje cell layer (PCL), and internal granule layer (IGL). GCPs are mitotically active in the outer EGL (oEGL) with maximal proliferation at P8 in response to the mitogen sonic hedgehog (SHH) (8). After multiple rounds of cell division, GCPs exit the cell cycle, move into the deeper layer of the EGL (premigratory layer, inner EGL [iEGL]), and differentiate into mature granule cells (GCs). GCs migrate radially along the Bergmann glial axons in the ML, passing the PCL that contains the soma of PCs and Bergmann glia, and form the IGL, the final position of GCs (7).

We have previously demonstrated that development of the cerebellum is altered in MCMV-infected mice, including globally altered morphogenesis (e.g., decreased cerebellar size, area, weight, and foliation) and changes in cortical structures (e.g., thicker EGL and decreased thickness of ML and IGL) (3, 9). Cerebellar hypoplasia in MCMV-infected animals is associated with reduced GCP proliferation in the EGL and delayed migration from the EGL to the IGL resulting in a paradoxically thickened EGL in infected mice (3). Because CNS infection in this model is focal without histologic evidence of necrosis and/or apoptosis of resident cells, it is unlikely that altered cerebellar development results from virus-induced cytopathology (3). However, MCMV infection induces a robust inflammatory response, including expression of interferon-stimulated genes and cytokines (e.g., IFIT1 and TNF-α), reactive gliosis, and recruitment of inflammatory monocytes/macrophages, neutrophils, NK cells, and CD8^+^ T cells into the brains of infected mice, suggesting that virus-induced host immune responses contributed to global and symmetric changes in the cerebellum (3, 10–14). Consistent with a mechanism of virus-induced immunopathology, treatment with corticosteroids or anti–TNF-α neutralizing antibodies partially corrected the dysmorphogenesis of the developing CNS, while minimally affecting virus replication in the CNS (9, 13, 15). In contrast, damage to the developing CNS following intracerebral inoculation of embryonic mice appeared directly attributable to virus replication and cytopathology during very early stages of neurodevelopment (16).

A variety of mechanisms can lead to impaired neurogenesis and the development of microcephaly and/or cerebellar hypoplasia (17). Infants or fetuses with Down syndrome can exhibit brain hypoplasia or microcephaly, including findings associated with reduced neurogenesis in the EGL of the cerebellum (18). An engineered murine model (Ts65Dn) of Down syndrome revealed that the GCP cell cycle was significantly delayed due to prolonged G1 and G2 phases with impaired neurogenesis attributed to decreased responsiveness to SHH during postnatal cerebellar development (19, 20). Similarly, multiple studies of the developing cerebral cortex have demonstrated that neural progenitor cell (NPC) proliferation and brain size are largely influenced by cell cycle length, with a strong correlation between G1 lengthening and NPC differentiation status (17, 21–24). Decreasing the length of G1 can lead to an inhibition of neurogenesis and expansion of progenitor pool, while lengthening of the G1-phase leads NPCs to transition from proliferative symmetric division to asymmetric neurogenic division (25–29). These studies support the hypothesis that the length of G1 is a critical determinant for cell differentiation during cortical development.

Although the cerebral cortex and the cerebellum share fundamental processes during development, the development of the cerebellum of rodents exhibits several features distinct from the developing cerebral cortex. In the developing cerebellum, GCPs are generated from the rhombic lip between E12.5 and E15.5, migrate to the cerebellar anlage forming the EGL, and continue to actively proliferate throughout the postnatal development until about P21. This process differs from proliferation and differentiation of NPCs in the developing cerebral cortex, which occurs during embryogenesis (30, 31). In addition, GCPs in the developing cerebellum predominantly undergo symmetric division regulated primarily by SHH, switching from nonterminal symmetric division to terminal symmetric division as development proceeds (32–35). However, the effect of insults such as virus-induced inflammation on cell cycle regulation of GCPs during postnatal cerebellar development have not been well characterized in in vivo models.

In the current study, we utilized established methodologies for quantifying cell cycle parameters to investigate the effect of inflammation on the reduced proliferation of GCPs in the developing cerebella of MCMV-infected mice. Our results demonstrate that MCMV-induced cerebellar inflammation leads to an increase in the length of G1 and S phases in GCPs, resulting in decreased GCP proliferation. In addition, lengthening of G1 and S phases was associated with premature exit of GCPs from the cell cycle, thus providing a mechanism for decreased cellularity of the IGL, altered cerebellar foliation, and cerebellar hypoplasia, all of which are characteristics of altered cerebellar development in MCMV-infected mice (3). Decreasing inflammation in this model by treatment with anti–TNF-α neutralizing antibody partially corrected cell cycle abnormalities in GCPs of MCMV-infected mice and normalized some of the cerebellar morphologic abnormalities, findings consistent with earlier studies (15). Thus, these findings potentially point to cell cycle dysregulation as mechanism for altered CNS development following CNS inflammation during neurodevelopment, informing the mechanism underlying altered brain development observed in fetuses and infants infected in utero with HCMV.

## Results

### MCMV replicates in the cerebellum and induces robust inflammatory response throughout postnatal development.

**Figure 1:**
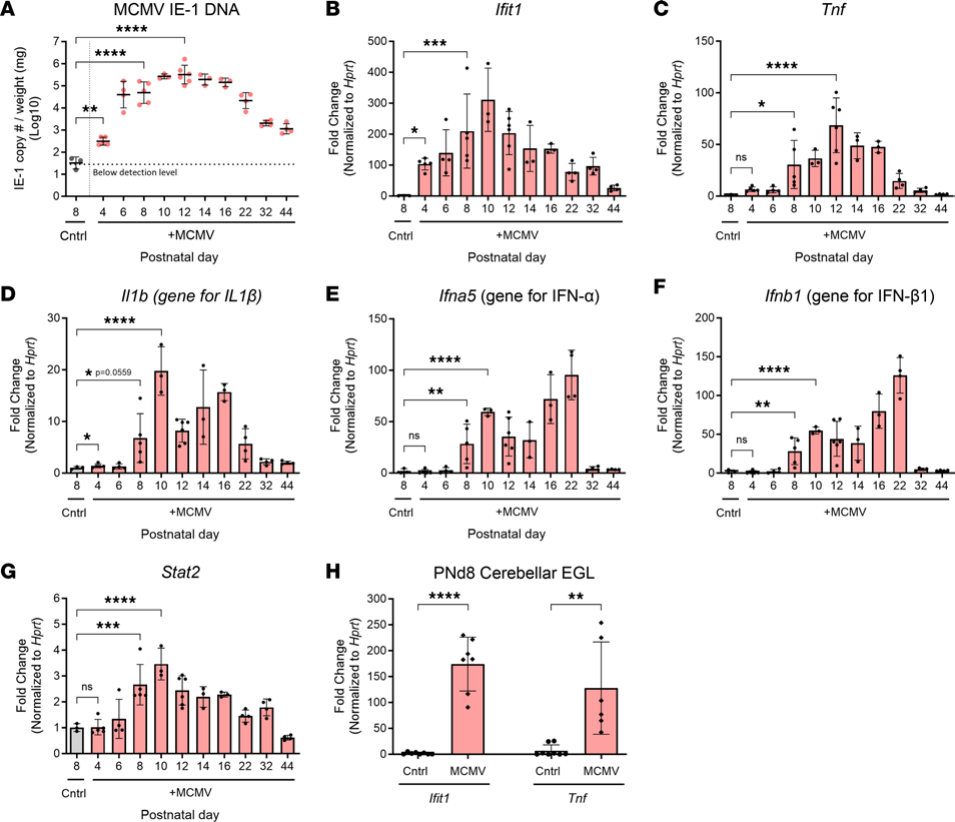
MCMV infection induces a robust inflammatory response during the postnatal period. DNA/RNA was extracted from cerebella of MCMV-infected mice at various time points (P4–P44) as described in Methods. (**A**) RT-PCR quantitation of MCMV DNA from cerebellum, with each data point representing genome copy number/mg of tissue. (**B**–**G**) Transcription of inflammatory mediators (*Ifit1*, *Tnf*, *Il1b* [gene for IL-1β], *Ifna5a* [gene for IFN-α], and *Ifnb1* [gene for IFN-β1]) and transcription factor (*Stat2*) quantified in the postnatal period and adulthood. *Hprt* was used as an internal control to normalize results, and fold change was calculated between cerebellar RNA from MCMV-infected and control animals. (**H**) Expression of *Ifit1* and *Tnf* were quantified by RT-PCR using RNA extracted from cerebellar EGL isolated by laser microdissection**.** A total of 3–6 cerebella were used for each experiment. The data are shown as mean ± SD. Each data point corresponds to a sample from an individual mouse. Statistical analyses were performed using 1-way ANOVA with Dunnett’s multiple-comparison test (main column effect) (**A**–**G**) or 2-tailed *t*-test (**H**). *P < 0.05; **P < 0.01; ***P < 0.001; ****P < 0.0001.

**Figure 2:**
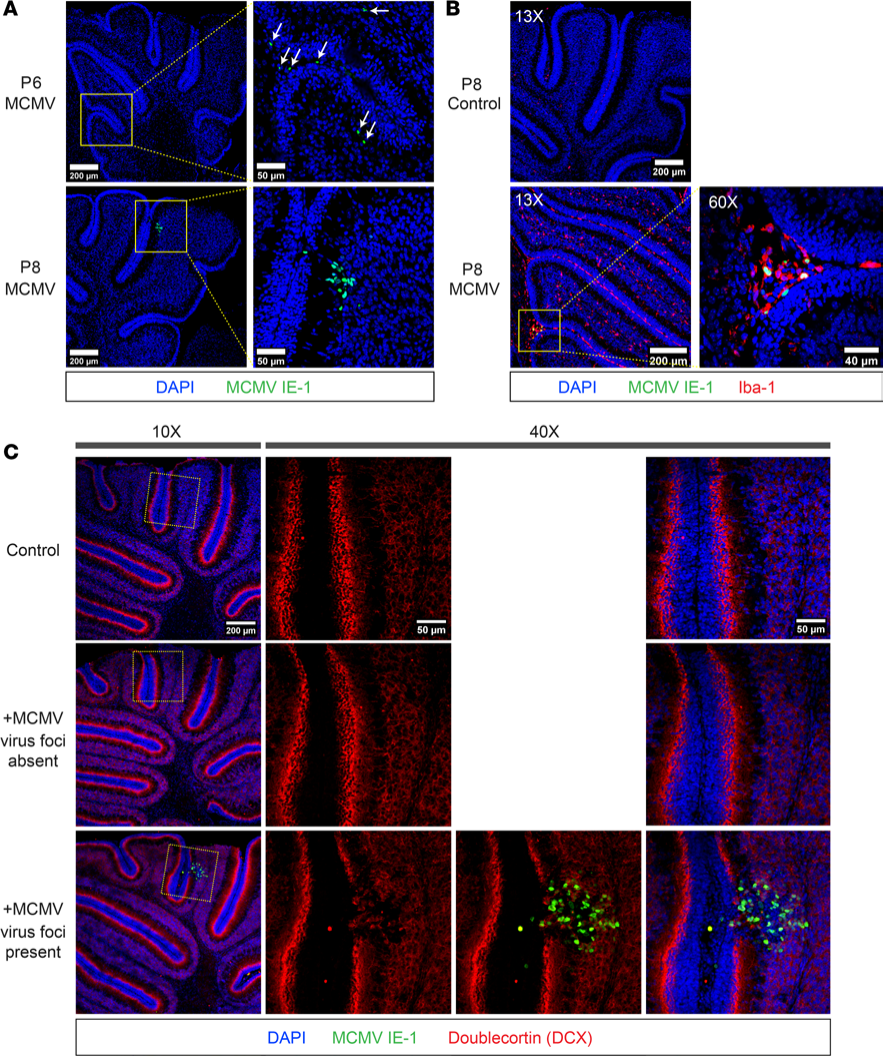
MCMV replicates in the cerebellum during the postnatal period. (**A**) Distribution of MCMV-infected cells in P6 (white arrow) and P8 cerebella detected with antibody reactive with MCMV IE-1 (pp89). Scale bar: 200 μm (left column) and 50 μm (right column). (**B**) Iba1^+^ mononuclear cells (red) in the cerebellum that also express MCMV IE-1 protein (green). Scale bar: 200 μm (13× images) and 40 μm (60× images). (**C**) P8 cerebellum double stained for MCMV IE-1 (green) and doublecortin (DCX) (red). DAPI (blue) was used to stain the nucleus. Scale bar: 200 μm (10× images) and 50 μm (40× images). Four cerebella were used per group.

Following i.p. inoculation of newborn mice with MCMV, MCMV DNA was detected in the brain as early as P4, reaching a peak at P12, and decreasing in subsequent time periods (Figure 1A). The gene expression of a subset of proinflammatory molecules, including an IFN-stimulated gene (*Ifit1*), type I interferons (*Ifna5* and *Ifnb1*; genes for IFN-α and IFN-β1), proinflammatory cytokines (*Tnf* and *Il1b*; genes for TNF-α and IL-1β), and an inflammation-associated transcription factor (*Stat2*), in cerebella from MCMV-infected mice followed similar kinetics in the postnatal period as MCMV DNA expression (Figure 1, B–G). These molecules were selected based on findings from genome-wide transcription studies done at several points during cerebellar development (3, 9, 15). To investigate potential sources of proinflammatory gene expression in the cerebellar cortex, we isolated cerebellar EGL from P8 uninfected and MCMV-infected mice by laser-capture microdissection (LMD). Cerebellar EGL from infected mice exhibited robust increases in both *Ifit1* and *Tnf* expression, suggesting that cells within the EGL of infected mice — including both resident and, potentially, cells infiltrating the EGL — contributed to the inflammatory response during infection even though this region of the cerebellum was not specifically targeted by the virus (Figure 1H). To further define the extent of MCMV infection in the cerebella of newborn mice, a monoclonal antibody reactive with the major immediate early-1 (IE-1) protein of MCMV (pp89) was used to identify infected cells (36). In P6 cerebellum, MCMV infection was detected in single cells; however, by P8, MCMV infection was identified as foci of infection in several regions of the cerebellum as well as in other areas of the brain, including frontal cortex and hippocampus as previously reported (Figure 2A) (3). MCMV infection was also occasionally detected in cells expressing Iba-1, a marker for activated brain microglia or infiltrating monocytes (Figure 2B). Lastly, the effect of foci of virus-infected cells on normal cerebellar morphology was determined by staining P8 brain sections with antibodies reactive with MCMV IE-1 and doublecortin (DCX), a microtubule-associated protein expressed in immature/mature differentiating and migrating GCs in the cerebellum (37). Areas of the cerebellum containing foci of virus-infected cells exhibited aberrant morphology of DCX**^+^** cells spanning the cerebellar cortex, whereas minimal morphologic alterations were present in regions of the cerebellum without virus-infected cells (Figure 2C). Consistent with previous observations, peripheral infection of newborn mice with MCMV led to virus infection in the cerebellum characterized by widely scattered foci of infection and the induction of robust inflammatory responses during cerebellar development (3, 9, 15).

### Cerebellar GCPs exhibit prolonged cell cycle length and increased cell cycle exit in MCMV-infected cerebella.

**Figure 3:**
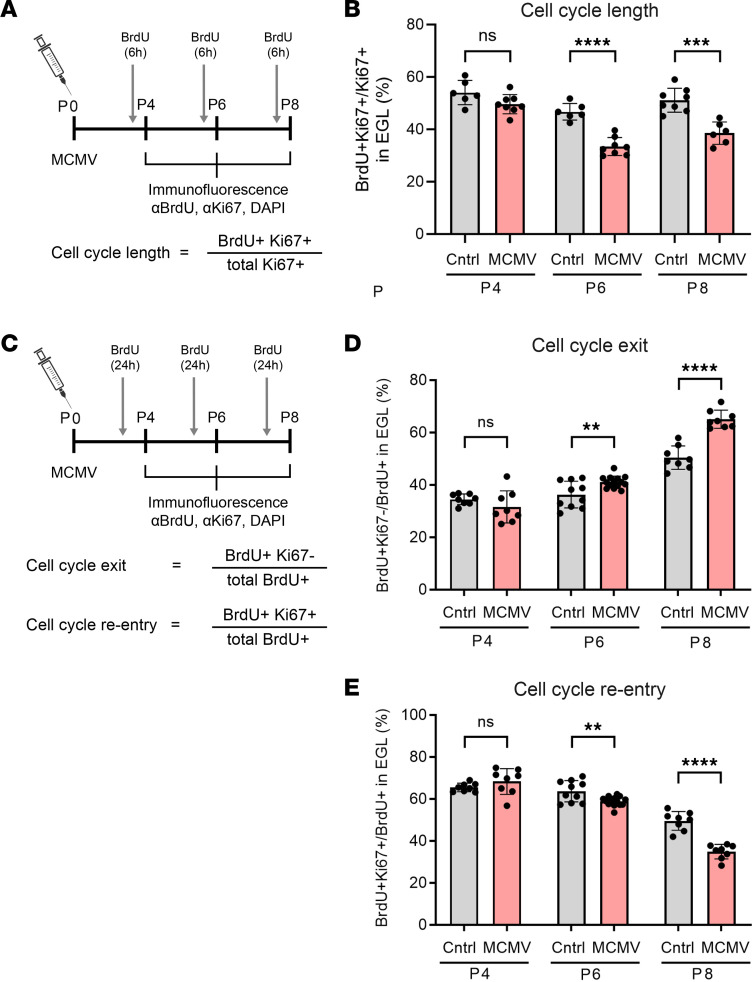
MCMV infection in newborn mice leads to longer cell cycle, increased cell cycle exit, and reduced cell cycle reentry of GCPs. (**A**) Schematic of 6-hour BrdU-incorporation protocol. (**B**) Cell cycle length was estimated as percentage of Ki67 and BrdU double positive cells in total Ki67^+^ cells (BrdU^+^Ki67^+^/total Ki67^+^ [%]) in EGL with a smaller percentage of double-positive cells indicating longer cell cycle. Representative images appear in Supplemental Figure 1. (**C**) Schematic of 24 hours BrdU-incorporation protocol. (**D**) Cell cycle exit defined as percentage of cells no longer in cell cycle defined by number of BrdU^+^Ki67^–^ cells to all cells labeled with BrdU (green) but not Ki67 (GCP cell cycle exit = BrdU^+^Ki67^–^/total BrdU^+^ in the EGL [%]). (**E**) Cell cycle reentry was defined as the percentage of cells that reentered the following cell cycle represented by ratio of BrdU^+^Ki67^+^ cell population to all cells labeled with BrdU (GCP cell cycle reentry = BrdU^+^Ki67^+^/total BrdU^+^ in the EGL [%]). (**C**–**E**) Please find representative images in Supplemental Figure 3. Data shown as mean ± SD, *n* = 3–5 mice/experimental group. Images of cerebellar fissure are represented as 2 data points. *P* values were calculated using 2-tailed *t* test. *P < 0.05; **P < 0.01; ***P < 0.001; ****P < 0.0001.

Despite the focal nature of MCMV infection in the brain, deficits in the cerebellar cortical development were global and, importantly, symmetric. MCMV infection has been associated with altered cerebellar cortical development, including decreased area and foliation, thinner IGL and ML, and a thicker EGL (3). In these previous studies, we observed decreased BrdU incorporation in GCPs in the EGL of infected mice, indicating that even though the EGL contained a greater number of cells, fewer were proliferating (3). These findings argue that the proliferation of GCPs in the EGL was delayed or perhaps incomplete and, as a result, delayed differentiation of GCPs into GCs and migration from EGL to IGL. To determine if altered GCP proliferation in infected mice was secondary to cell cycle dysregulation, we assessed the cell cycle length, cell cycle exit, and cell cycle reentry of GCPs (38). First, we confirmed previous findings that the percentage of cells in cell cycle (Ki67^+^ cells) in cerebella from MCMV-infected and control mice were comparable at 3 time points (P4, P6, P8) (Supplemental Figure 1, A and C; supplemental material available online with this article; https://doi.org/10.1172/jci.insight.175525DS1). Conversely, the percentage of BrdU^+^ cells was decreased in MCMV-infected cerebella as early as P6, with a greater reduction in BrdU^+^ cells in cerebella from P8 mice (Supplemental Figure 1, A and B) (3, 9, 15). The reduced proliferative capacity of GCPs in infected cerebella was also shown by fewer total mitotic cells as detected by phospho-histone H3 (pHH3) expression in cells in the EGL (Supplemental Figure 2). The discrepancy in results from 2 proliferation markers, BrdU and Ki67, suggested that there was a block or delay of cell cycle progression in GCPs in MCMV-infected mice. GCP cell cycle length was estimated by calculating the labeling index (LI) as a percentage of BrdU**^+^**Ki67**^+^** cells in total Ki67**^+^** cells, with a smaller percentage arguing that the cell cycle is longer relative to GCPs from control mice (Figure 3A). In cerebella from infected mice, cell cycle length of GCPs was not affected at P4 but was significantly prolonged at P6 and P8, as indicated by the decreased LI (Figure 3B). Together, these data suggest that GCPs in the MCMV-infected mice were cycling more slowly than GCPs in control mice and that lengthening of the cell cycle in GCPs in infected mice could account for the overall reduction in the number of GCs in the cerebellar cortex of infected mice.

We next determined if GCP cell cycle exit and reentry was altered in MCMV-infected mice as compared with control mice. Control and MCMV-infected mice were pulse labeled with BrdU 24 hours prior to harvesting at P4, P6, and P8, and fixed brain sections were analyzed for cells expressing BrdU and Ki67 (Figure 3C). The percentage of cells that exited the cell cycle was defined by the proportion of BrdU**^+^**Ki67***^–^*** cells in the total population of BrdU**^+^** cells (Figure 3C). Cell cycle reentry was quantified by calculating the percentage of cells that reentered cell cycle, defined as the proportion of BrdU**^+^**Ki67**^+^** cell in the total population of BrdU**^+^** cells during the 24-hour interval (Figure 3C) (38). Compared with controls, we observed a significant increase in the percentage of GCPs that exited the cell cycle as well as a lesser number of cells reentering the cell cycle in EGL from P6 and P8 but not from P4 in MCMV-infected mice (Figure 3, D and E). These observations were consistent with altered kinetics of the cell cycle of GCPs in infected mice and provided additional evidence that a reduced number of GCPs progressed through the cell cycle, thus accounting for a decreased number of GCs and cerebellar hypoplasia in infected mice.

Following proliferation, GCPs exit the cell cycle in the oEGL, differentiate into immature GCs, move into the deeper layer of the EGL, the iEGL, and then migrate along Bergmann glial fibers to the IGL (39). If GCs fail to move from the oEGL to the iEGL, subsequent migration into the IGL is eliminated (7). We previously reported that increased cellularity of the cerebellar EGL is associated with delayed migration of GCPs from EGL to IGL in MCMV-infected mice (3). To further characterize abnormalities associated with altered proliferation and premature cell cycle exit of the GCPs in the EGL of infected mice, we investigated migration of GCPs within the EGL of mice pulse labeled with BrdU. Immunofluorescence using anti-BrdU and anti-Ki67 antibodies revealed a significant reduction in the number of BrdU**^+^** GCPs migrating from the oEGL to the iEGL on P6 and P8 but not on P4 in cerebella of MCMV-infected mice (Supplemental Figure 3). We confirmed this result using pulse-chase labeling of MCMV-infected and control mice with an alternative thymidine analog IdU for 18, 22, 25, 28, 32, 36, and 40 hours prior to sacrifice at P8 and determined whether migration of IdU^+^ GCPs was blocked or delayed as defined by detection of IdU**^+^** GCPs in the iEGL. The iEGL was demarcated by immunostaining with antibody against transiently expressed axonal glycoprotein (TAG-1), a contactin-related adhesion molecule (40, 41). We found maximum numbers of IdU**^+^** GC cells in the iEGL at 32 hours in control mice and 40 hours for GCs of MCMV-infected mice, suggesting that MCMV infection resulted in a significant delay in movement of GCs from the oEGL to the iEGL (Supplemental Figure 4). These results argue that MCMV infection dysregulated GCP cell cycle progression, increased cell cycle exit, and delayed GC migration within the EGL of the cerebellar cortex.

### GCPs in MCMV-infected mice have increased cell cycle length due to an increase in G1 and S phases during postnatal development.

**Figure 4:**
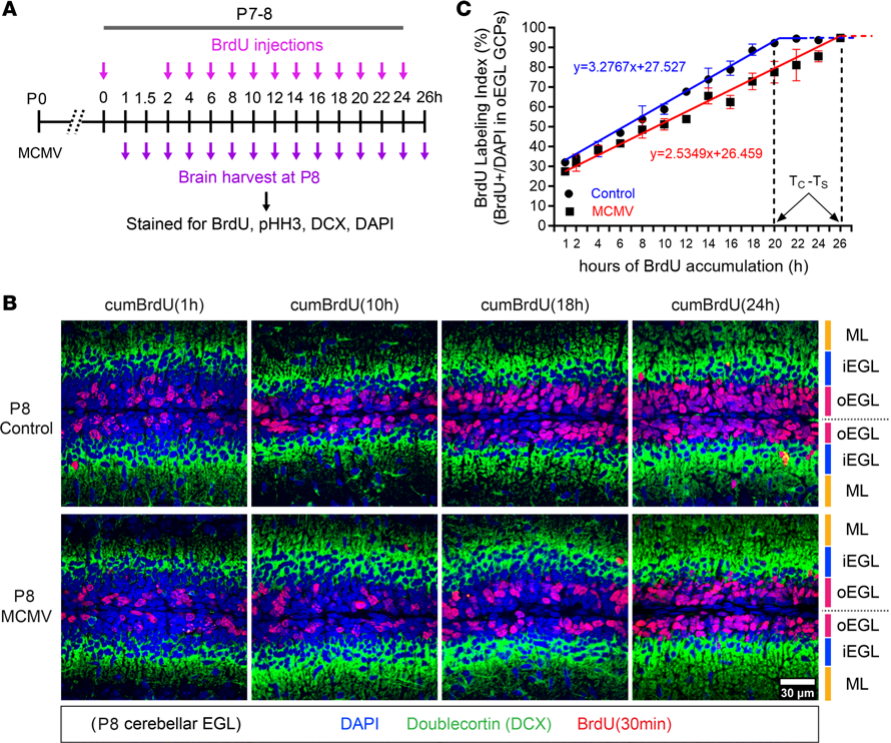
Prolonged GCP cell cycle during MCMV infection is due to the lengthening of G1 and S phases. (**A**) Schedule of in vivo cumulative BrdU labeling protocol to estimate length of each phase of cell cycle of GCPs in the cerebellar EGL. (**B**) Representative images of brain sections stained for BrdU (red), DCX (labeling the iEGL; green), and DAPI (nuclei staining; blue) from control and MCMV-infected mice exposed to cumulative BrdU for the indicated time. In MCMV-infected cerebellum, fewer GCPs incorporated BrdU at time points are shown (1, 10, 18, and 24 hours) compared with control mice. Scale bar: 30 μm. (**C**) BrdU^+^ GCPs in the oEGL of the cerebella were quantified (BrdU LI) at time points and plotted against duration of BrdU exposure to estimate cell cycle parameters. The BrdU LI was used to determine the duration of the cell cycle (T_C_) and time required to complete S phase (T_S_). Refer to Table 1 for cell cycle length (total cell cycle length, G1 phase, and S phase). Data are shown as mean ± SD, *n* = 3–4 mice/experimental group of the cerebellum.

**Figure 5:**
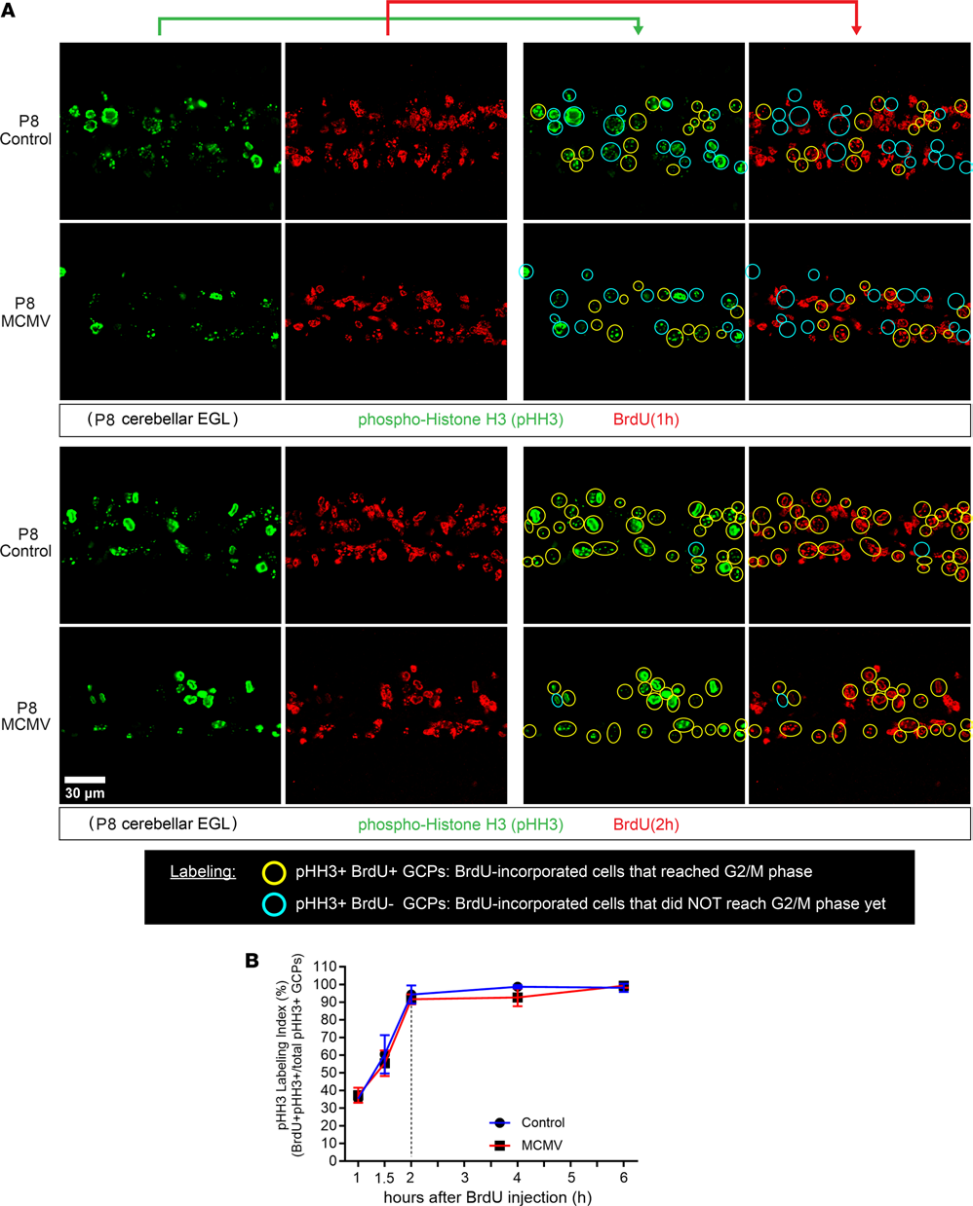
Prolonged GCP cell cycle during MCMV infection is not due to the lengthening of G2/M phase. (**A**) The duration of G2/M phase (T_G2+M_) was determined by single-dose BrdU labeling for 1, 1.5, and 2 hours and stained for BrdU (red) and for pHH3 (green) to define cells in G2/M phase; see Figure 4A for the schedule of the in vivo cumulative BrdU labeling protocol. pHH3^+^BrdU^–^ cells (light blue open circle) are pHH3^+^ cells not in S phase at the time of BrdU injection. BrdU^+^pHH3^+^ cells (yellow open circle) are cells that incorporated BrdU in the S phase and reached G2/M phase. Scale bar: 30 μm. (**B**) pHH3 labeling index (pHH3 LI = BrdU^+^pHH3^+^/total pHH3^+^ GCPs in the EGL [%]) was determined. Please refer to Table 1 for cell cycle length (G2/M phase). Data are shown as mean ± SD, *n* = 4–6 mice/experimental group of the cerebellum. *P* values were calculated using 2-tailed *t* test. *P < 0.05; **P < 0.01; ****P < 0.0001.

Previous studies have suggested that cell cycle kinetics are closely linked to cell cycle exit and neuronal differentiation (42). Overexpression of Cyclin D1/Cdk4 in NPCs in the developing murine cerebral cortex shortened G1 and inhibited neurogenesis, whereas lengthening of G1 by inhibition of Cyclin D1/Cdk4 promoted neurogenesis (27). Thus, increasing the duration of G1 is sufficient to induce cell cycle exit and differentiation of NPCs. Based on these findings, we utilized in vivo cumulative BrdU labeling to estimate total cell cycle length (T_C_) as well as the length of different phases of the cell cycle of GCPs in control and MCMV-infected mice. Beginning on P7, BrdU was administered to control and MCMV-infected mice every 2 hours from 0 to 24 hours, and cerebella were harvested on P8 (Figure 4A) (43). Brain sections were stained for BrdU and DCX, a marker for differentiated GCs in the iEGL, in order to exclude postmitotic cells from the analyses (44). BrdU**^+^** GCPs in the oEGL were quantified at each time point to generate a BrdU LI (ratio of BrdU**^+^** GCPs to total cell number). In agreement with our findings that GCPs were cycling in both infected and control mice, the GCPs in the oEGL reached the maximum BrdU LI in both groups; however, the time required for the BrdU LI to reach a plateau was longer for GCPs in the MCMV-infected mice (26 hours) compared with control mice (20 hours) (Figure 4, B and C). To determine the T_C_ and duration of S phase (T_S_), the duration of BrdU exposure was plotted against BrdU LI and the best-fitted line was calculated. T_C_ and T_S_ were increased by 8.06 hours and 2.02 hours, respectively, in GCPs of MCMV-infected mice (Figure 4 and Table 1). The duration of G2/M phase (T_G2+M_) in P8 mouse GCPs was determined by administering a single-pulse BrdU injection followed by harvesting brains after 1, 1.5, and 2 hours and quantifying the number of pHH3**^+^** and BrdU**^+^** GCPs (Figure 4). The mitotic LI was estimated by the percentage of BrdU^+^pHH3^+^ cells of the total pHH3^+^ cells, allowing an estimate of the minimum time required for BrdU**^+^** GCPs to enter G2/M (pHH3**^+^**). The maximum mitotic LI of GCPs was reached within 2 hours in both control and MCMV-infected cerebella (Figure 5 and Table 1). The duration of G1, derived by subtracting the duration of S and G2/M phases (T_S+G2+M_) from the T_C_ increased by 6.04 hours in GCPs of MCMV-infected mice compared with GCPs in control mice (Table 1).

**Figure 6:**
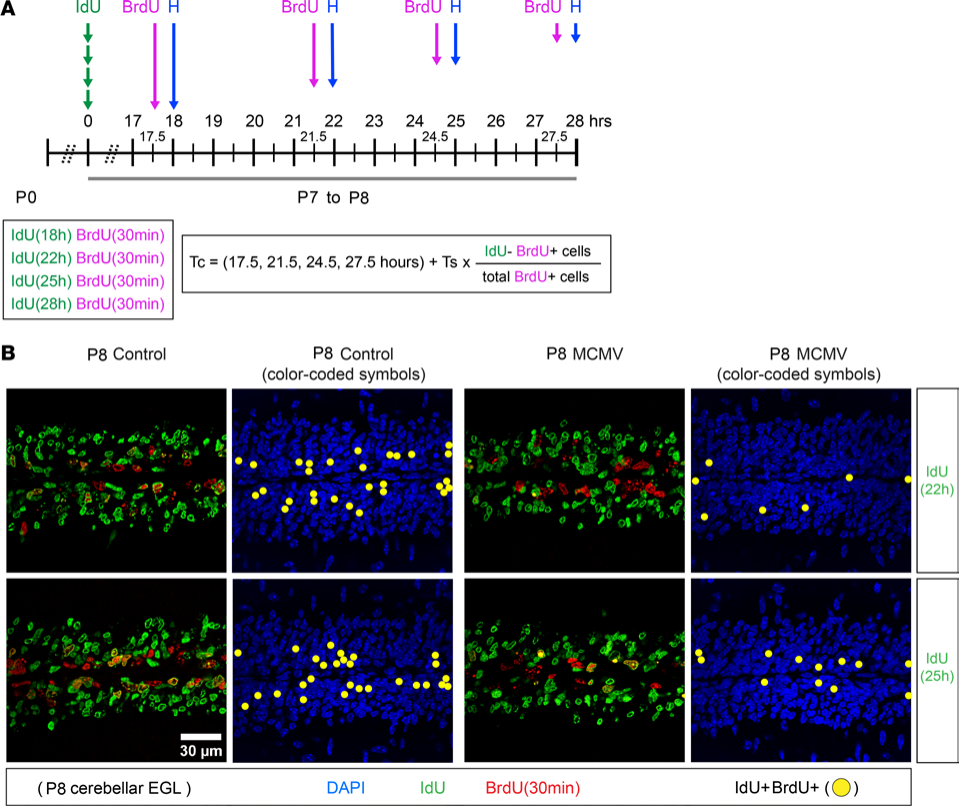
BrdU-IdU sequential labeling confirms a longer G1 phase of the cell cycle in MCMV-infected mice. (**A**) Schematic of the IdU-BrdU dual-labeling protocol to determine total cell cycle length (T_C_). IdU was injected on P7, followed by BrdU injection at 17.5, 21.5, 24.5, and 27.5 hours after IdU injection on P8. Brains were then harvested 30 minutes later. IdU^–^BrdU^+^ and total BrdU^+^ GCPs were quantified to determine the T_C_. (**B**) Representative images and images with color-coded symbols of cerebellar EGL indicate a lesser number of GCPs reentered S phase of the subsequent cell cycle (IdU^+^BrdU^+^ cells; yellow solid circle) in MCMV-infected mice compared with control mice during interval between IdU and BrdU injections (22 or 25 hours). Cerebellar sections were stained for IdU (green), BrdU (red), and DAPI (blue). Scale bar: 30 μm. Refer to Table 1 for T_C_. T_C_ was approximately 8.7 hours longer in GCPs in MCMV-infected mice compared with control mice.

**Figure 7:**
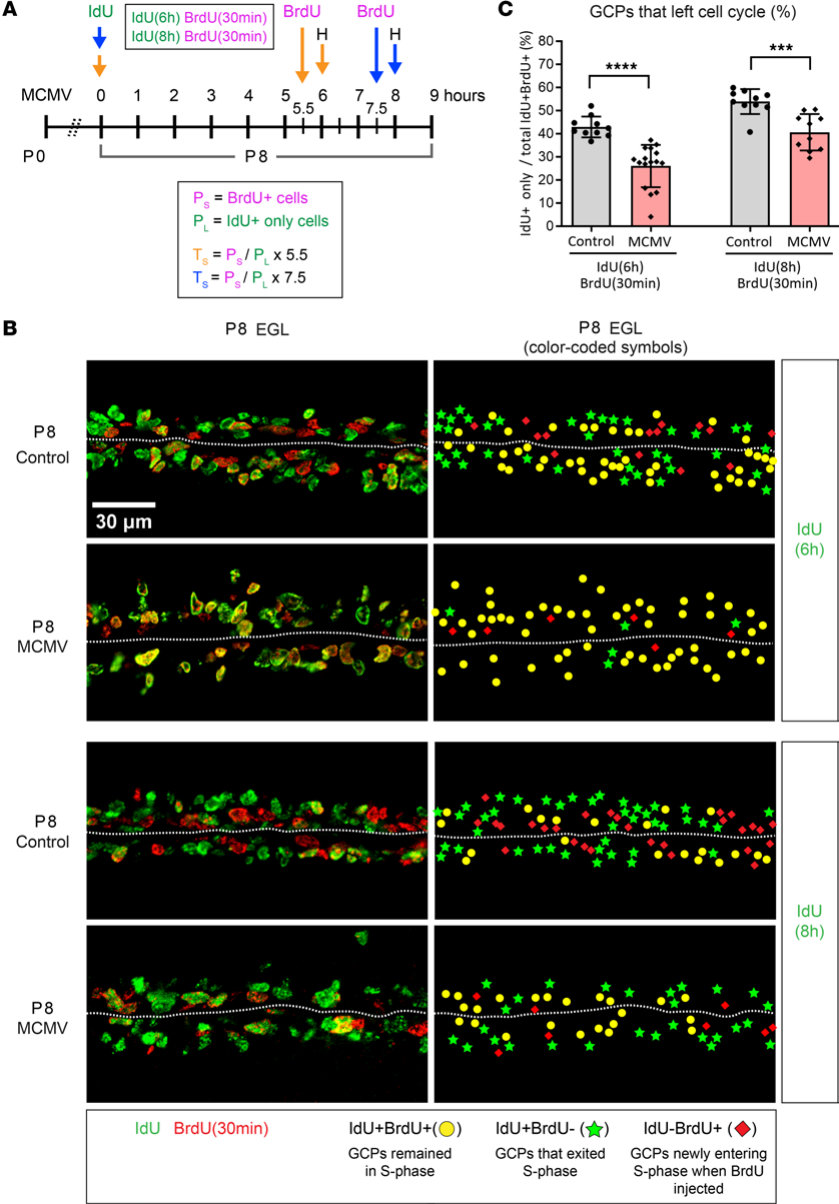
BrdU-IdU sequential labeling confirms a longer S phase of the cell cycle in MCMV-infected mice. (**A**) Schematic of IdU-BrdU dual-labeling protocol to measure the length of S phase (T_S_). IdU was injected, followed by BrdU injections at 5.5 and 7.5 hours after IdU injection. Brains were then harvested 30 minutes after the BrdU injection on P8. (**B**) Representative images with color-coded symbols of cerebellar EGL indicate reduced number of cells that exited S phase in MCMV-infected mice during the inter–injection intervals of 6 or 8 hours. IdU^+^BrdU^+^ GCPs are population that remained in S phase (yellow solid circle), and IdU^+^BrdU^–^ GCPs are population that left S phase (green solid star). Scale bar: 30 μm. (**C**) Percentage of GCPs that left S phase was quantified as IdU^+^/total IdU^+^BrdU^+^ GCPs in the EGL. Refer to Table 1 for cell cycle length (S phase). T_S_ was approximately 5.2 hours longer in GCPs in MCMV-infected mice compared with control mice. Data are shown as mean ± SD, *n* = 4–6 mice/experimental group of the cerebellum. Images of cerebellar fissure are represented as 2 data points. *P* values were calculated using 2-tailed *t* test. ***P < 0.001; ****P < 0.0001.

Conventional analyses of cumulative BrdU labeling experiments assume that proliferating cells are distributed uniformly throughout the cell cycle and that the number of cells increases with steady state dynamics predicted from models of asymmetric cell division (43, 45). However, studies from live cell imaging of ex vivo whole mount cerebella have reported that, at later stages of cerebellar development (P10), approximately 4% of GCPs progress through asymmetric division while most GCPs go through terminal symmetric division (~73%) or nonterminal symmetric division (~22%) (33). Thus, the increased duration of T_C_ and T_S_ in GCPs derived from cumulative BrdU labeling in cerebella from infected mice was confirmed using an assay based on sequential IdU-BrdU double labeling. Cumulative BrdU labeling estimated that T_C_ – T_S_ in control and MCMV-infected mice were 20 and 26 hours, respectively (Table 1). Therefore, to determine T_C_ by sequential IdU-BrdU labeling, we used an interval between IdU and BrdU injections that included the duration of T_C_ – T_S_ determined by cumulative BrdU labeling. This was to ensure the detection of 2 cell populations: IdU**^+^** cells that entered S phase of the following cell cycle (IdU**^+^**BrdU**^+^** cells) and those that did not reenter or had exited S phase of the following cell cycle (IdU**^+^**BrdU**^–^** cells). Mice were pulse labeled with IdU for 18, 22, 25, or 28 hours and injected with BrdU 30 minutes prior to harvesting brains on P8 (Figure 6A). Reduced numbers of GCPs entered S phase of the following cycle (IdU**^+^**BrdU**^+^** cells) in the MCMV-infected mice, indicating GCPs from infected animals were exiting cell cycle and not reentering (Figure 6B). Sequential IdU-BrdU labeling revealed that T_C_ was approximately 8.7 hours longer in GCPs in MCMV-infected cerebella compared with control cerebella, confirming results from studies using cumulative BrdU labeling (Table 1). To measure T_S_, P8 mice were pulse labeled with IdU for 6 or 8 hours and injected with BrdU 30 minutes prior to harvesting brains at P8 (Figure 7A). T_S_ was determined by counting the number of GCPs in S phase (IdU**^+^**BrdU**^+^** cells) and was compared with the number of cells that exited S phase (IdU**^+^**BrdU**^–^** cells) during the interval between injections (Figure 7B). GCPs from MCMV-infected mice displayed a significantly reduced number of cells that exited the S phase between 6 and 8 hours, with T_S_ being approximately 5.2 hours longer than GCPs of control mice (Figure 7, B and C, and Table 1). The duration of G1, derived by subtracting T_S+G2+M_ from T_C_, increased by 3.5 hours in GCPs in MCMV-infected mice compared with that of control mice (Table 1). MCMV infection increased the length of the cell cycle of GCPs in postnatal mice secondary to an increased duration of both G1 and S phases but not G2/M.

### GCP cell cycle signaling pathway is disrupted during MCMV infection.

To elucidate mechanisms underlying the decrease in GCP proliferation and lengthening of G1 and S phases following MCMV infection, we first determined if the SHH signaling pathway was altered in cerebella from infected mice. SHH is responsible for GCP proliferation in the cerebellar cortex during postnatal development (46). By performing in situ hybridization and analyzing total cerebellar RNA and protein, we found that both *Shh* mRNA and SHH protein were decreased in cerebella of MCMV-infected mice (Supplemental Figure 5). To establish that reduction in SHH expression as measured in total cerebella led to changes in the SHH signaling pathway in GCPs, we quantified expression of key downstream components of SHH, including PTCH1, SMO, GLI1, GLI2, and MYCN, of GCPs in the EGL. Unexpectedly, in both control and MCMV-infected mice, we observed comparable mRNA expression of SHH downstream components in RNA isolated from the EGL obtained by laser microdissection as well as by in situ hybridization (Supplemental Figure 6, A–C). Similarly, no significant differences in the protein expression of downstream components of the SHH pathway were noted when assayed in GCPs isolated from the cerebella of control or MCMV-infected mice (Supplemental Figure 6, D and E). Thus, even though SHH appeared to be decreased in the cerebella of MCMV-infected animals, downstream effectors of the SHH signaling pathway in GCPs appeared intact, suggesting that altered SHH expression was not the primary factor affecting GCP proliferation, specifically cell cycle length, in infected animals.

**Figure 8:**
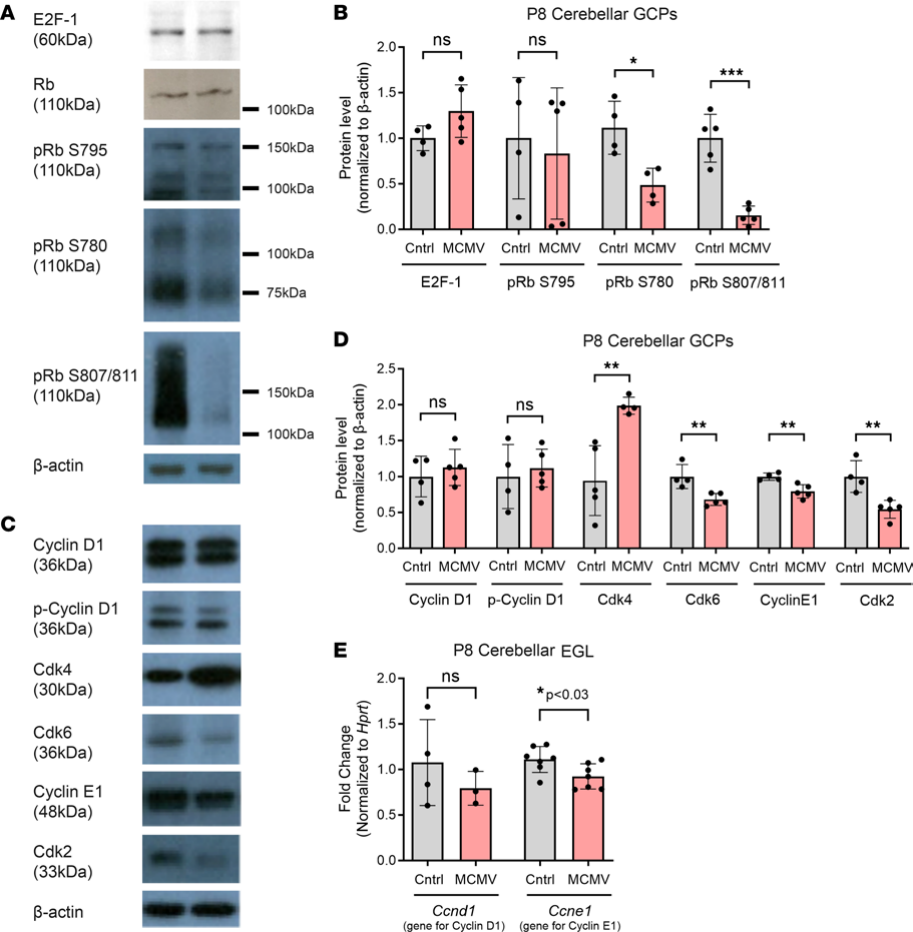
MCMV infection in newborn mice alters phosphorylation of Rb and levels of cell cycle proteins in GCPs. (**A**–**D**) The expression of cell cycle proteins was quantified in GCPs isolated from P8 control and MCMV-infected cerebella by immunoblotting. β-Actin was used as internal control. Four to 5 samples (4 cerebella pooled for each sample)/experimental group were used for immunoblot analysis. (**A** and **B**) Expression of total Rb, phosphorylated Rb (pRb), and E2F-1 were quantified. Levels of total Rb, pRb S795, and E2F-1 were unaltered; however, pRb S780 and pRb S807/811 were significantly decreased in GCPs from MCMV-infected mice compared with control mice. Note immunoblots probed for pRb S807/811 were overexposed to allow detection of this form of Rb in GCPs from infected mice. (**C** and **D**) GCPs from MCMV-infected cerebella showed differential regulation of Cdk4 and Cdk6 while Cyclin D1 or p-Cyclin D1 were unaltered. Cyclin E1 and Cdk2 levels were reduced in GCPs from MCMV-infected mice cerebella. (**E**) Transcript levels of *Ccnd1* (gene for Cyclin D1) and *Ccne1* (gene for Cyclin E1) were quantified by RT-PCR using RNA extracted from cerebellar EGL isolated by laser microdissection. *Ccnd1* and *Ccne1* transcript levels correlated with the protein levels from isolated GCPs. *n* = 3–7 mice/experimental group were used for RT-PCR analysis. Data are shown as mean ± SD. *P* values were calculated using 2-tailed *t* test. *P < 0.05; **P < 0.01; ***P < 0.001.

The retinoblastoma (Rb) protein blocks entry into the S phase by binding to E2F transcription factors, which regulate progression through G1 and G1/S transition. Phosphorylation of Rb by cyclin-dependent kinases release E2F transcription factors from Rb and enable progression from G1 to the S phase of the cell cycle (47–49). Thus, we examined the effect of MCMV infection on Rb, Cyclin D1, and Cdk4/6 in GCPs isolated from P8 control and MCMV-infected cerebella (50). While we observed similar protein levels of total Rb protein, we detected significantly reduced levels of Rb phosphorylated at Ser780 (S780) and S807/811 in GCPs from MCMV-infected cerebella relative to GCPs from control mice (Figure 8, A and B). In contrast, the protein levels of Rb phosphorylated at S795 and E2F-1 in MCMV-infected GCPs were comparable with those in GCPs from control mice (Figure 8, A and B). The protein levels of both Cyclin D1 and p-Cyclin D1 (Thr 286) were also unaltered in both groups, and we found no difference in the mRNA level of *Ccnd1* (gene for Cyclin D1) from the cerebellar EGL obtained by laser microdissection (Figure 8, C–E). However, Cdk4/6 were differentially regulated with a significant increase in Cdk4 and a significant decrease in Cdk6 protein levels in the MCMV-infected GCPs (Figure 8, C and D). Finally, the amounts of Cyclin E1 and Cdk2 were lower in GCPs isolated from MCMV-infected mice compared with control mice (Figure 8, C and D), consistent with the decreased mRNA level of *Ccne1* (gene for Cyclin E1) from the cerebellar EGL obtained by laser microdissection (Figure 8E). Reduced levels of Cyclin E1 and Cdk2 in infected animals could limit hyperphosphorylation of Rb, thus slowing the release of E2F and delaying G1/S transition. These data are consistent with the lengthened G1 and S phases observed in GCPs from MCMV-infected mice.

### Treatment with TNF-NAb normalizes the prolonged G1 and S phases in GCPs of MCMV-infected mice.

**Figure 9:**
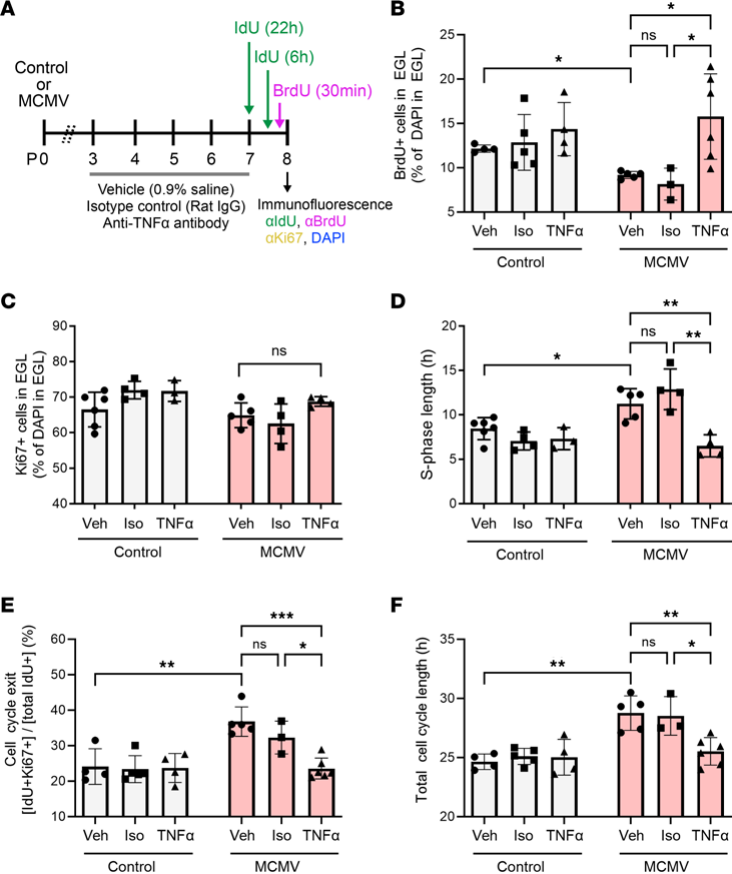
Treatment with TNF-NAb normalizes cell cycle abnormalities in MCMV-infected mice. (**A**) Schematic representation of IdU-BrdU dual-labeling protocol in MCMV-infected mice and control mice treated with vehicle (Veh), isotype control antibody (Iso), or TNF-α neutralizing antibody (TNF-NAb). Fixed brain sections were stained for IdU, BrdU, Ki67, and DAPI to measure cell cycle parameters as described in previous figures. (**B** and **C**) Brain sections from mice treated with BrdU for 30 minutes were analyzed to measure cell proliferation by quantifying BrdU^+^ (**B**) and Ki67^+^ (**C**) GCPs in the EGL. (**D**) Mice were labeled with IdU for 6 hours, followed by BrdU injection 30 minutes prior to harvesting brains at P8 to estimate the length of S phase of GCPs in the EGL. (**E** and **F**) Mice were pulse labeled with IdU for 22 hours and injected with BrdU 30 minutes prior to harvesting brains at P8 to estimate cell cycle exit (**E**) and total cell cycle length (**F**). Data shown as mean ± SD, *n* = 4–6 mice/experimental group for immunofluorescence. Each data point corresponds to cerebellar EGL from an individual mouse. *P* values were calculated by using 1-way ANOVA with Tukey’s multiple-comparison test. *P < 0.05; **P < 0.01; ***P < 0.001.

Previously, we have shown that treatment of MCMV-infected newborn mice with either an antiinflammatory corticosteroid or an anti–TNF-α neutralizing antibody (TNF-NAb) normalized morphometric abnormalities in the cerebella of infected mice, thus providing direct evidence that virus-induced inflammatory responses were, in part, responsible for altered cerebellar development in MCMV-infected mice (9, 15). We extended these findings in the current study by exploring the effect of TNF-NAb on cell cycle abnormalities in infected mice. Infected and control newborn mice were treated once daily with vehicle, isotype control antibody, or TNF-NAb on P3–P7, and the effect on the cell cycle in GCPs determined on P8 (Figure 9A). The effect of TNF-NAb treatment on the T_S_ was estimated by injection of mice with IdU for 6 hours followed by injection with BrdU 30 minutes prior to harvesting brains (Figure 9A). Consistent with previous studies, decreased BrdU incorporation was observed in the cerebella of vehicle-treated or isotype control antibody–treated MCMV-infected mice, whereas treatment of infected mice with TNF-NAb normalized BrdU incorporation to levels comparable with control mice (Figure 9B and Supplemental Figure 1, A and B) (15). The percentage of Ki67^+^ GCPs remained unchanged in all 3 treatment groups of MCMV-infected mice compared with control mice (Figure 9C and Supplemental Figure 1, A and C). We also observed an extended S phase in GCPs of MCMV-infected mice receiving vehicle or isotype control antibody, while the length of S phase was normalized in TNF-NAb–treated MCMV-infected mice (Figure 9D). P7 mice were injected with IdU for 22 hours and later with BrdU 30 minutes prior to harvesting brains to determine the proportion of cells exiting cell cycle and cell cycle length (Figure 9A). TNF-NAb treatment of MCMV-infected mice normalized the proportion of cells exiting cell cycle as well as the T_C_ of GCPs, while isotype control antibody treatment had no significant effect on cell cycle regulation (Figure 9, E and F). Lastly, although these data indicate that TNF-α–regulated inflammatory responses induced by MCMV contributed to the perturbations in the cell cycle of GCPs in the cerebella of MCMV-infected mice, it is unlikely that a single proinflammatory molecule could fully account for the altered cerebellar development observed in MCMV-infected mice.

## Discussion

Newborn infants infected in utero with HCMV can exhibit a variety of structural brain abnormalities and can present clinically with a spectrum of neurodevelopmental sequelae (51–54). Small animal models, including guinea pigs, rats, and mice, have been developed to be used in the study of CNS disease associated with congenital HCMV infections. Murine models have relied almost exclusively on direct MCMV inoculation of the brain of both embryos and newborn animals (55, 56). In this report, we used i.p. inoculation of newborn mice, a route resulting in hematogenous spread of MCMV to the CNS leading to cerebellar hypoplasia characterized by decreased cerebellar size, area, and foliation — findings that have also been described in HCMV-infected infants (3, 53). Changes in the cerebellar cortex included increased thickness of the EGL and decreased thickness of ML and IGL (3). Other animal models mimicking various examples of altered brain development (genetic or infectious agents) have identified several mechanisms underlying microcephaly, including increased apoptosis, reduced proliferation, and/or premature differentiation of NPCs (17, 20, 57–59). In this model, an increased number of apoptotic cells was not detected in cerebella from infected mice as compared with control mice, indicating that increased cell death did not account for reduced cerebellar size; however, reduced proliferation and delayed migration of GCPs in the cerebellar cortex was noted (3, 9, 15). In the current study, we have demonstrated that virus-induced inflammation leads to dysregulation of the cell cycle and altered cerebellar development in MCMV-infected mice.

We determined that the cell cycle length of GCPs in the infected mice was longer than in control mice but, importantly, not arrested. Relative to the control samples, there was a decreased number of BrdU**^+^** GCPs while the number of Ki67**^+^** GCPs was unchanged in the cerebellar EGL of infected mice, indicating that while cells entered the cell cycle, there was a delay in cell cycle progression in infected mice (Figure 3B and Supplemental Figure 1). We quantified the duration of G1, S, and G2/M phases of the cell cycle and demonstrated that reduced proliferation of GCPs in infected mice resulted from lengthening of both G1 and S phases. Altered GCP proliferation could be secondary to direct viral damage to the GCPs or indirect effects such as virus-induced inflammatory responses. Direct viral damage is an unlikely explanation because virus-infected cells were only found in widely scattered foci in the cerebellum, a finding inconsistent with the symmetric and global dysmorphogenesis of the cerebellum observed in this model. Moreover, foci of infected cells were rarely observed in the EGL. Thus, the prolonged cell cycle length of GCPs is more likely due to virus-induced CNS inflammation, a mechanism consistent with normalization of cerebellar cortical development following treatment with corticosteroids or TNF-NAb (9, 15). We confirmed these earlier results and demonstrated that inhibiting TNF signaling normalized cell cycle abnormalities in GCPs in infected mice (Figure 9). A potential mechanism consistent with prolonged S phase in GCPs of MCMV-infected mice is that inflammation leads to DNA damage response (DDR) pathways. Prior studies have shown that DNA damage slows S phase progression (60). Thus, it is possible that GCPs slowed in S phase in order to repair DNA damage secondary to MCMV-induced inflammation. TNF-α has been suggested as an indirect cause of DDR through the production of ROS and altered mitochondrial function, with evidence that TNF-α–induced mitochondrial ROS can lead to cytotoxicity (61, 62). Because *Tnf* transcripts were increased approximately 30-fold in cerebella of MCMV-infected mice on P8 and continued to increase through P12 (Figure 1C), it is possible that MCMV-induced TNF-α expression could have induced mitochondrial ROS in the GCPs of MCMV-infected mice and lead to genomic instability. However, we observed only rare cleaved caspase-3^+^ GCPs in the cerebella of MCMV-infected mice, suggesting it was unlikely that there was substantial DNA damage present in GCPs of infected mice. More recently, Kvestak, et.al., reported that IFN-γ also contributed to altered cerebellar morphogenesis similar to the phenotypes we have described in MCMV-infected newborn mice, including increased thickness of the EGL compared with control animals (63). In this study, IFN-γ produced by infiltrating NK/ILC1 were suggested to contribute to increased thickness of the EGL of the cerebellum secondary to IFN-γ–driven expression of SHH (63). In contrast to this study that utilized the total cerebellum, in the current study, the transcription of downstream targets of SHH remained unaltered when RNA was analyzed from isolated GCPs and cells of the EGL isolated by laser microdissection. Thus, while we detected decreased levels of *Shh* transcripts and SHH protein consistent with alterations in the proliferative program of the GCPs of infected animals, the downstream SHH signaling pathway within the GCPs appeared intact, arguing against altered SHH signaling (Supplemental Figure 5) (46, 64, 65). While it is possible that the magnitude of the decrease in SHH expression was insufficient to alter downstream signal amplification, it should be noted that there appeared to be some evidence of increased transcription of *Mycn*, a downstream target of *Shh* (Supplemental Figure 6C). Thus, dysregulation in the SHH signaling pathway and the thickened EGL phenotype in infected mice in this model remains to be further defined. Lastly, insulin-like growth factor (IGF) has also been implicated as a potent mitogen for GCPs during postnatal development and synergizes with the signaling pathway of SHH (66). Although changes in IGF expression remain a potential explanation for our findings, our previous transcriptomic studies indicate that there was not a notable change in IGF expression in cerebella from infected mice as compared with control mice (3).

Interestingly, despite an intact SHH signaling pathway in GCPs of the infected mice, cell cycle proteins regulating G1-phase and G1/S transition were altered, including the phosphorylation of Rb and expression of Cyclins and Cdks. Normally, Rb protein acts as a suppressor by binding to E2F transcription factors and inhibit their activity. During cell cycle, Cdks phosphorylate Rb protein causing conformational change that weakens its binding affinity for E2F. This phosphorylated Rb releases E2F transcription factors, allowing them to translocate into the nucleus and activate the expression of genes necessary for cell cycle progression from G1 to S phase (49, 67, 68). Refinement of this original model argues that cell cycle progression during mid-G1 phase first requires monophosphorylation of Rb/p105 at residues including Ss780, Ss795, or S807/811 by Cyclin D-Cdk4/6 complexes. Subsequently, Cyclin E-Cdk2 kinase activity hyperphosphorylates Rb, leading to the release of E2F transcription factors and E2F translocation into the nucleus to regulate gene expression necessary for S-phase entry and DNA replication (48, 69, 70). Thus, the phosphorylation state of Rb can be viewed as an indirect measure of cyclin-associated kinase activity. In our studies, Rb phosphorylation at S795 was similar in MCMV-infected and control mice; however, phosphorylation at S780 and S807/811 was significantly reduced in GCPs from infected mice, raising the possibility that additional phosphorylation events and, ultimately, release of E2F transcription factors occurred with reduced efficiency. This interpretation was in agreement with decreased Cyclin E1 and Cdk2 protein expression in the GCPs of infected mice (Figure 8). In contrast to reduced levels of Cyclin E1 and Cdk2, we did not detect changes in the Cyclin D1 or p-Cyclin D1 levels in GCPs from infected mice relative to control samples, while levels of Cdk4 and Cdk6 were increased and decreased, respectively. Because the activities of Cdk4 and Cdk6 are redundant with regard to Rb phosphorylation, the changes in the steady state levels of the Cdk catalytic subunits could have had a negligible effect on its modification. Unaltered levels of Rb phosphorylated on S795 and expression of Rb phosphorylated S780 or S807/811, albeit at significantly reduced levels in GCPs of MCMV-infected mice compared with control mice, argued that the Cyclin D-Cdk4/6 complexes maintained some degree of kinase activity in GCPs of infected mice. These results suggest that Rb phosphorylation is altered but still present in GCPs of MCMV-infected mice and further support our findings that progression through G1-phase is delayed but not blocked in GCPs during MCMV infection.

Overexpression of Cyclin D1 and Cyclin E1 in NPCs in the mouse cerebral cortex resulted in a shortened duration of G1-phase of the cell cycle, promoted cell cycle reentry, and inhibited neuronal differentiation (28). In contrast, lengthening of G1 phase can cause proliferating cells to prematurely exit cell cycle, lead to neurogenesis, and subsequently result in the reduction of the overall population size of NPCs of the developing cerebral cortex (21, 27, 29, 71–73). Our results are consistent with the findings in NPCs in that lengthening of the G1 phase was accompanied by an increase of cell cycle exit of GCPs in infected animals and decreased cell cycle reentry compared with control animals.

Collectively, our results argue that virus-induced inflammation and not direct virus cytopathic effects drive MCMV neuropathogenesis during this period of cerebellar development (Supplemental Figure 7). These findings together with further characterization of cellular responses to MCMV-induced inflammation during neurodevelopment could help identify therapeutic targets and, in turn, contribute to the development of more effective treatments of CNS infections that include indirect effects of infection such as virus-induced inflammation.

## Methods

### Sex as a biological variable

Our study examined both male and female mice; however, sex was not considered a biological variable because the study was conducted primarily on neonatal mice at P8 or younger. Visually identifying the sex of fetal and neonatal mice is not reliably feasible. In addition, sex has not been shown to be a determinant in MCMV-induced CNS disease or CNS disease associated with HCMV infection.

### Animals and MCMV infection

Pathogen-free BALB/c mice (The Jackson Laboratory) were housed under pathogen-free conditions. MCMV virus (Smith strain repaired reading frame M128) stocks (74) were propagated in M2-10B4 bone marrow stromal cells (ATCC, CRL-1972) and harvested at peak cytopathologic effect, and aliquots of viral stocks were stored at –80°C (74, 75). Infectious virus titers were quantified by plaque assays in mouse embryonic fibroblast (MEF) (76, 77). Male and female newborn mouse pups were infected with 500 plaque forming unit (PFU) of MCMV diluted in phosphate-buffered saline (PBS). Mice were injected i.p. within 12 hours of birth (3). For tissue harvest, mice were euthanized by CO_2_ asphyxiation followed by cardiac perfusion with 1× PBS.

### Quantitation of virus genome copy number and gene expression

Details can be found in the Supplemental Methods.

### Immunofluorescence and in situ hybridization

Details can be found in the Supplemental Methods.

### Immunoblot

Description of reagents and conditions for these methods are standard and have been published in previous manuscripts (13, 15) and are available in the Supplemental Methods.

### TNF-α neutralizing antibody (TNF-NAb) treatment

Pups were treated daily (P3–P7) by i.p. injection with InVivoMAb rat IgG1 Isotype control anti-trinitrophenol (anti-TNP) (In VivoMAb rat IgG1 Isotype control; anti-TNP; BioXcell; BE0290; clone TNP6A7) or InVivoMAb anti–mouse TNF-α neutralizing antibody (tat IgG1, clone XT3.11, BioXCell) at 500 μg/mouse/day diluted in PBS. MCMV-infected and uninfected mice that received only an injection of sterile 1× PBS (vehicle) served as controls. On P8, mouse pups were sacrificed and exhaustively perfused with 1× PBS (3). Organs including brains were harvested and prepared for appropriate downstream assays.

### Laser microdissection (LMD)

P8 mice were sacrificed and perfused as described above. After stripping of the meninges, brains were harvested and embedded in optimum cutting temperature (OCT) compound. Brain sections (15 μm) were cut, adhered to PEN membrane-coated glass slides (Leica Microsystems Inc.), and frozen at –80°C. Prior to LMD, slides were thawed at room temperature for 1 minute, fixed for 1 minute in 70% ethanol, washed in RNase-free water, and stained with 0.2% (w/v) cresyl violet for 30 seconds. Sections were then washed in water and dehydrated by graded series of ethanol (70%, 95%, 100%) for 1 minute each and air dried for 5 minutes. A Leica LMD6 microscope system (Leica Microsystems) was used to perform microdissection of the cerebellar EGL and dissected regions collected in 0.5 mL tube in GTC lysis buffer containing β-mercaptoethanol. The collection tubes were vortexed and centrifuged (16,110g for 5 minutes at room temperature) repeatedly then placed directly in dry ice or stored at –80°C. RNA was isolated with RNeasy Mini Kit (Qiagen) with on-column DNase digestion. cDNA was synthesized and reverse transcription PCR (RT-PCR) was performed (Supplemental Methods).

### In vivo deoxyuridine labeling

Details on the preparation of BrdU and IdU can be found in the Supplemental Methods.

#### Cumulative labeling.

GCP cell cycle phase durations were determined using cumulative BrdU labeling (43, 78, 79). Briefly, MCMV-infected mice and noninfected control mice received repeated injections of BrdU every 2 hours for up to 24 hours (0, 2, 4, 6, 8, 10, 12, 14, 16, 18, 20, 22, and 24 hours). After the respective cumulative BrdU pulse labeling for each time point, pups were sacrificed at P8 (1, 1.5, 2, 4, 6, 8, 10, 12, 14, 16, 18, 20, 22, 24, and 26 hours after first BrdU injection) (Figure 4A). Brains were then harvested, fixed with 4% paraformaldehyde (PFA), sectioned, and stained for BrdU, DCX, and pHH3 (S10) (Supplemental Table 1). DCX is expressed in differentiated GCs in the iEGL and used to identify and exclude postmitotic cells from the analyses (44). The BrdU LI is defined as the percentage of BrdU^+^ cells per total number of cells in oEGL, which increased over time. Growth fraction is determined when the BrdU LI reaches a plateau with constant percentage (Table 1) (43). Mitotic cells in the oEGL were stained for pHH3, and mitotic LI (mitotic LI; pHH3 LI) was quantified to determine the duration of G2/M phase (19, 80–82). Calculations used for determination of the BrdU LI and pHH3 LI are shown below. Analysis of the cumulative BrdU experiment provided T_C_ and provided each phase of the cell cycle (T_G1_, T_S_, and T_G2+M_) in GCPs in the oEGL of cerebella from control and MCMV-infected mice.


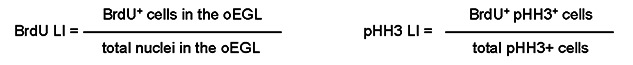


#### Dual labeling.

To confirm the T_C_ and T_S_ from cumulative BrdU labeling experiment, we performed sequential IdU-BrdU dual labeling of GCPs. To estimate T_C_, pups were given a single i.p. injection of IdU for 18, 22, 25, or 28 hours followed by a single injection of BrdU 30 minutes prior to harvesting brains on P8 (Figure 6A). Brain sections were then stained for IdU and BrdU. The T_C_ was calculated by quantifying cells in S phase only during the second injection (IdU^–^BrdU^+^ cells) and total BrdU^+^ cells, which not only includes (IdU^–^BrdU^+^ cells) but also includes cells initially in S phase that incorporated IdU and reentered S phase of the following cell cycle (IdU^+^BrdU^+^ cells) (Figure 6A) (83). The formula used to calculate T_C _is shown below. The number of IdU^+^BrdU^+^ cells, representing GCPs that initially incorporated IdU and entered S phase of the following cell cycle, was quantified to assess the number of GCPs that reentered the next cell cycle (Figure 6B).


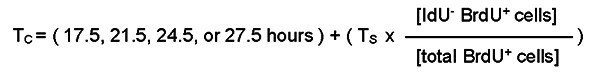


To determine the T_S_, P8 pups were given a single i.p. injection of IdU at 6 or 8 hours, followed by a single i.p. injection of BrdU 30 minutes prior to harvesting on P8 (Figure 7A). Brain sections were stained for IdU and BrdU, and the formula used to calculate T_S_ is shown below (83–89). T_S_ was determined by quantifying GCPs that initially incorporated IdU and stayed in S phase (IdU^+^BrdU^+^ cells) compared with GCPs that initially incorporated IdU but did not incorporate BrdU secondary to exit from S phase (IdU^+^BrdU^–^ cells) during the 6- or 8-hour interval between injections (Figure 7A).


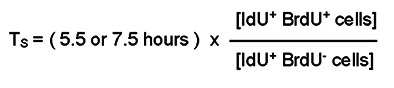


### Confocal imaging, cell counting, and statistical analysis

Cerebellar images were acquired using the Olympus FV1000 confocal microscope and Olympus Fluoview FV10 ASW 4.2 software (Olympus). Identical magnification and laser power were used within the same experiment to acquire images of the cerebellar EGL. Subsequently, Fiji (ImageJ) was used to process confocal images and manually quantified GCPs by using the cell counter plugin (ImageJ > Plugins > Analyze > Cell counter). GCPs were quantified along the entire span of the EGL within the acquired images (164 μm).

### GCP isolation

Primary GCPs were isolated from cerebella as previously described (90, 91). Briefly, 4 cerebella were pooled from P8 control and MCMV-infected BALB/c mice and dissected in Ca^2+^- and Mg^2+^-free HBSS (Thermo Fisher Scientific) supplemented with glucose. The meninges were stripped and cerebella were trypsinized (Worthington Biochemical Corp.) in 37°C for 15 minutes followed by trituration to make a single-cell suspension in HBSS/DNase/soybean trypsin inhibitor solution (MilliporeSigma). Cells were filtered through a 70 μm cell strainer and were carefully decanted on top of 4% BSA/HBSS and centrifuged at 70*g* for 5 minutes at 4°C to filter out large cells. To obtain a fraction enriched for GCPs, the cell suspension was loaded on a Percoll (GE Healthcare) gradient of 35% and 65% and centrifuged at 1,800*g* for 10 minutes at room temperature. GCPs were recovered from the 35%/65% interface, washed in HBSS/glucose solution, pelleted, and stored in –80°C until used for downstream experiments.

### Statistics

All statistical analyses were performed using Prism 6 (GraphPad). The 2-tailed Student’s *t* test was used to compare statistical significance between 2 sample groups, noninfected and MCMV-infected mice. The Shapiro-Wilk’s test was used to analyze data sets for normality. Comparisons of multiple groups were subjected to ordinary 1-way ANOVA with Tukey’s post hoc multiple-comparison test for data with equal variances or otherwise by Dunn’s comparisons test to determine significance across treatment groups. One-way ANOVA with Dunnett’s multiple-comparison test was employed to compare the treatment groups against a single control group. Data are reported as mean ± SD. Values were statistically significant at *P* < 0.05.

### Study approval

All animal protocols (APN9351) and procedures were approved by IACUC and Animal Resource Program (ARP) of the University of Alabama at Birmingham and were in compliance with *Guide for the Care and Use of Laboratory Animals* (National Academies Press, 2011).

### Data availability

All data supporting the findings of this study are available within the paper and its supplemental materials. The values for all data points in graphs are available in the Supporting Data Values file. Additional data are available from the corresponding author upon request.

## Author contributions

CYWS contributed conceptualization, data curation, formal analysis, investigation, methodology, project administration, software, validation, writing of the original draft, and writing review and editing. ML contributed investigation and data curation. SJ contributed conceptualization and resources. VS contributed data curation. WJB contributed conceptualization, review and editing of the manuscript, project administration, supervision, resources, and funding acquisition.

## Supplementary Material

Supplemental data

Unedited blot and gel images

Supporting data values

## Figures and Tables

**Table 1:**
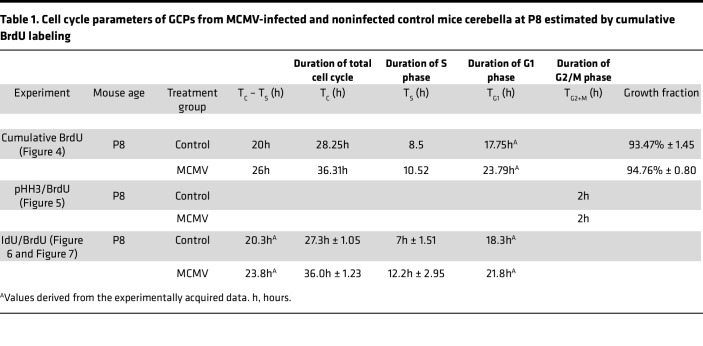
Cell cycle parameters of GCPs from MCMV-infected and noninfected control mice cerebella at P8 estimated by cumulative BrdU labeling

## Version 1 06/10/2024

Electronic publication
