# Supplementary material for: Cytomegalovirus infection lengthens the cell cycle of granule cell precursors during postnatal cerebellar development
Source: JCI Insight. 2024 Jun 10;9(11):e175525. doi: 10.1172/jci.insight.175525 (PMC11382886; doi:10.1172/jci.insight.175525)
Supplement: Supplemental data [file jciinsight-9-175525-s184.pdf]

Supplemental Materials

Cytomegalovirus infection Lengthens the Cell Cycle of Granule Cell Precursor during Postnatal Cerebellar Development

Cathy Yea Won Sung[1,6], Mao Li[2], Stipan Jonjic[4,5], Veronica Sanchez[2], and William J Britt[1,2,3]

[1]Department of Microbiology, [2]Department of Pediatrics, [3]Department of Neurobiology, University of Alabama at Birmingham, School of Medicine, Birmingham, Alabama, USA

[4]Department of Histology and Embryology, [5]Center for Proteomics, Faculty of Medicine, University of Rijeka, Rijeka, Croatia.

[6]Laboratory of Hearing Biology and Therapeutics, National Institute on Deafness and Other Communication Disorders (NIDCD), NIH, Bethesda, Maryland, USA

Address correspondence to: William J. Britt, Department of Pediatrics, University of Alabama at Birmingham, Children's Harbor Bldg, room 107, Birmingham, AL 35294, USA. Phone: 205-638-2619; Email: wbritt@uabmc.edu.

Conflict of interest: The authors have declared that no conflict of interest exists.

Table of Content

Supplemental Methods

Quantitation of virus genome copy number and gene expression

Immunofluorescence and *in situ* hybridization

Western blot

Supplemental Figures

Supplemental Figure 1

Supplemental Figure 2

Supplemental Figure 3

Supplemental Figure 4

Supplemental Figure 5

Supplemental Figure 6

Supplemental Figure 7

Supplemental Table

Supplemental Table 1

Supplemental References

Supplemental Methods

In vivo deoxyuridine labeling (Preparation of BrdU and IdU)

Mice were injected i.p. with 150mg/kg body weight of thymidine analogs of 5-bromo-2-deoxyuridine (BrdU) or 5-Iodo-2-deoxyuridine (IdU) (Sigma Aldrich, St. Louis, MO). BrdU (25mg/ml) was dissolved in sterile 0.9% saline and incubated at 55°C for 15 minutes (1). Soluble BrdU was cooled to room temperature and aliquots were stored at -20°C. BrdU precipitates in thawed aliquots were redissolved at 55°C on dry heat block for 5-10 min and cooled to room temperature before use. IdU (70mg/ml) was dissolved in 0.2N NaOH/0.9% saline, adjusted to pH 9, and aliquots were stored in -20°C. IdU was further diluted in 0.9% saline to make 10mg/ml for injection into mice.

Quantitation of virus genome copy number and gene expression

Dissected cerebella were harvested at various time points and DNA and RNA were extracted from cerebella using DNeasy Blood & Tissue Kits (Qiagen, Germantown, MD) and E.Z.N.A Total RNA kit (Omega Bio-tek, Norcross, GA) with minor modifications in the manufacturer's protocol. Quantitative PCR (qPCR) was performed to detect MCMV immediate early-1 (IE-1) gene exon 4 from total DNA using forward primer (5'-GGC TCC ATG ATC CAC CCT GTT A-3'), reverse primer (5'-GCC TTC ATC TGC TGC CAT ACT-3') and probe (5'-AGC CTT TCC TGG ATG CCA GGT CTC A-3') labeled with FAM and TAMRA. A standard curve was generated from dilutions of plasmid containing IE-1 exon 4 by qPCR using TaqMan Gene Expression master mix (ThermoFisher Scientific, Waltham, MA) with samples run in duplicate on StepOne Plus Real-Time PCR system (Applied Biosystems, Life Technologies, Foster City, CA). Viral genome copy numbers were expressed as log(10) genome copies per milligram (mg) of tissue.

For quantitative reverse transcription PCR (RT-PCR) assays, cDNA from total RNA was synthesized with Invitrogen Superscript III First strand synthesis kit (Thermo Fisher Scientific, Waltham, MA). RT-PCR was performed using the same reagents and system as qPCR for MCMV DNA detection. TaqMan Gene Expression master mix was used with primers including *18S* (Mm03928990_g1), *Hprt* (Mm00446968_m1), *Ifit1* (Mm00515153_m1), *Tnf* (Mm00443258_m1), *Il1b* (Mm01336189_m1), *Ifna5* (Mm00833976_s1), *Ifnb1* (Mm00439552_s1), *Stat2* (Mm00490880_m1), *Shh* (Mm00436528_m1), *Ccnd1* (Mm00432359_m1; gene for Cyclin D1), and *Ccne1* (Mm01266311_m1; gene for Cyclin E1) (Life technologies, Foster City, CA). *18S* or *Hprt* were used as internal control genes and fold changes for experimental groups were expressed as $2^{-\Delta\Delta Ct}$ normalized to control group values.

Immunofluorescence and *in situ* hybridization

Animals were sacrificed and cardiac perfused with 1X PBS. Brains were then harvested and post-fixed in 4% PFA overnight at 4°C. Tissues were cryoprotected in 15% sucrose/PBS overnight followed by 30% sucrose/PBS for approximately 48 hours at 4°C. Subsequently, cryoprotected brains were incubated in 30% sucrose/OCT (1:1) solution for 2-3 hours at room temperature, embedded in OCT medium (Electron Microscopy Sciences, Hatfield, PA), and snap frozen in 2-methylbutane/dry ice. Eight-micron (8μm) brain sections were cut using a

cryostat, sections were dried overnight at room temperature, and either stained the day of processing or stored in -80°C.

For immunostaining, brain sections were rehydrated in PBS and incubated in blocking buffer (PBS, 0.3% TritonX-100, 2% normal goat serum) for 3-4 hours at room temperature followed by overnight incubation at 4°C with primary antibodies (Supplemental Table 1) diluted in blocking buffer. Incubation with primary antibodies was followed by a 2-hour incubation in species/isotype-matched secondary antibodies conjugated with FITC, TRITC (SouthernBiotech, Birmingham, AL), or Alexa Fluor 594 (ThermoFisher Scientific, Wiltham, MA) at room temperature. Hoechst dye (Hoechst 33342; ThermoFisher Scientific, Wiltham, MA) was used as nuclear stain. After immunostaining, brain sections were mounted with Vectashield (Vector Laboratories; Burlingame, CA) on glass slides.

For detection of $BrdU^+$ or IdU^+ cells, rehydrated brain sections were treated with 2N HCl for 20 minutes at 37°C and incubated in borate buffer (0.1M boric acid and 12.5mM sodium borate in distilled water) for 10 minutes at room temperature to neutralize brain sections. Subsequent downstream procedures were identical to immunostaining protocol described above.

Anti-TAG-1 immunostaining (Iowa Hybridoma bank, clone 4D7) utilized brain tissue from animals perfused with 1X PBS followed by 4% PFA cardiac perfusion. This second perfusion was essential for TAG-1 immunostaining to visualize staining in both the ridge/lobes and fissures of cerebellar folia. Subsequently, brains were harvested and post-fixed in 4% PFA overnight at 4°C. All other downstream procedures were identical to the immunofluorescence staining procedures described above.

Fluorescence *In situ* hybridization (FISH) was performed as described previously (2). Probe constructs for *Gli1*, *Mycn*, and *Shh* were generated by Drs. A.L. Joyner (Memorial Sloan Cancer Center, New York, NY, USA), D.H. Rowitch (University of Cambridge, Trumpington, Cambridge, UK), and A.P. McMahon (University of Southern California, Los Angeles, CA, USA), respectively. All three probes were generously provided by A.L. Joyner. Digoxigenin (DIG)-labeled RNA probes were transcribed *in vitro* using a DIG RNA labeling kit (Roche Applied Science, Basel, Switzerland) and brain sections hybridized with gene-specific antisense RNA probes. Sense transcript was used as a negative control. *Shh* mRNA detection was coupled with staining for calbindin to visualize Purkinje cells. Detection of *Gli1* and *Mycn* mRNA were coupled with protein staining for doublecortin (DCX) that stains iEGL to localize *Gli1* and *Mycn* transcripts in the oEGL.

Western blot

Samples from isolated GCPs were extracted by homogenization in RIPA buffer (50mM Tris-HCl pH7.5, 150mM NaCl, 1mM EDTA, 0.1% SDS, 1% NP-40, 0.5% Na-deoxycholate) containing Halt protease/phosphatase inhibitors (ThermoFisher, Waltham, MA). Homogenates were sonicated (10sec sonicate/1min on ice/10sec sonicate), incubated on ice for 30 min-1 hour, and then centrifuged at 13,200rpm for 10 min at 4°C. Total protein was quantified using BCA assay (Pierce/ThermoFisher, Waltham, MA) and equivalent amounts of proteins (30µg per sample) were loaded on a 9% SDS-polyacrylamide gel. The gels were then transferred onto a 0.45µm nitrocellulose membrane. Blotted membranes were blocked for at least 1 hour in 5% non-fat milk in TBS-t (10mM Tris-HCl, NaCl 150mM, pH 8.0)-Tween20 (0.1%) and incubated overnight with primary antibodies (Supplemental

Table 1). Following washing, membranes were incubated at room temperature for 2-3 hours with HRP-conjugated anti-rabbit, anti-mouse, or anti-rat secondary antibodies (Southern Biotech, Birmingham, AL) and developed using Western Lightning Plus-ECL (PerkinElmer, Waltham, MA). Densitometry was performed using Fiji (ImageJ, NIH, Bethesda, MD), and quantity of proteins were normalized to β-actin. Immunoblotting utilizing some antibodies was carried out by horizontally cutting nitrocellulose membrane at a specific molecular weight to enable immunological detection of proteins migrating markedly differently in an individual gel due to the difference in protein mass. This approach was taken secondary to limited amounts of sample and the number of membranes required for the experiments. In addition, this approach allowed detection of different targets from the same sample while limiting the background generated when antibodies from different species were applied to a single filter. Repeated stripping of membranes was considered but pilot experiments provided unsatisfactory results secondary to residual background.

Supplemental Figures

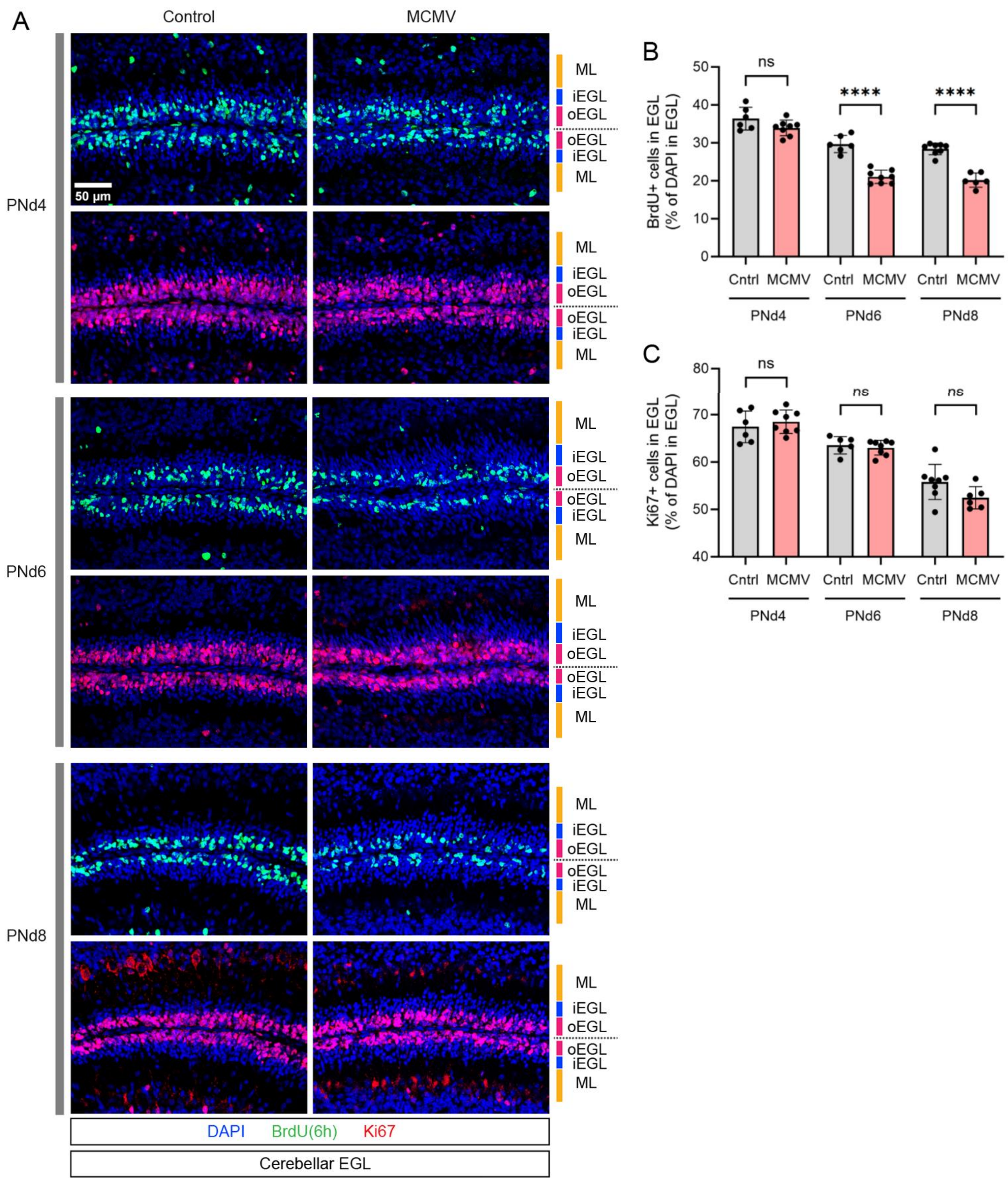

Supplemental Figure 1. MCMV infection is associated with a decrease in cerebellar GCP proliferation. (A-C) Representative images and quantification of BrdU^{+} (green) and Ki67^{+} (magenta) GCPs at PNd4, 6, and 8 in the EGL of non-infected control and MCMV-infected mice cerebella. Please find the schematic diagram illustrating the 6-hour BrdU-incorporation protocol in Figure 3A. Scale bar: 50 µm. **(B)** The percentage of BrdU^{+} cells was decreased in the EGL of MCMV-infected mice at PNd6 and PNd8. **(C)** The percentage of Ki67^{+} cells from MCMV-infected mice from PNd6 and PNd8 were comparable to non-infected control mice**.** Data are shown as mean ± SD, n=3-5 mice/experimental group. Each data point corresponds to the individual cerebellar EGL. Images of cerebellar fissure are represented as two data points. P-values were calculated using two-tailed t-test (*p<0.05; **P<0.01 ; ****p<0.0001).

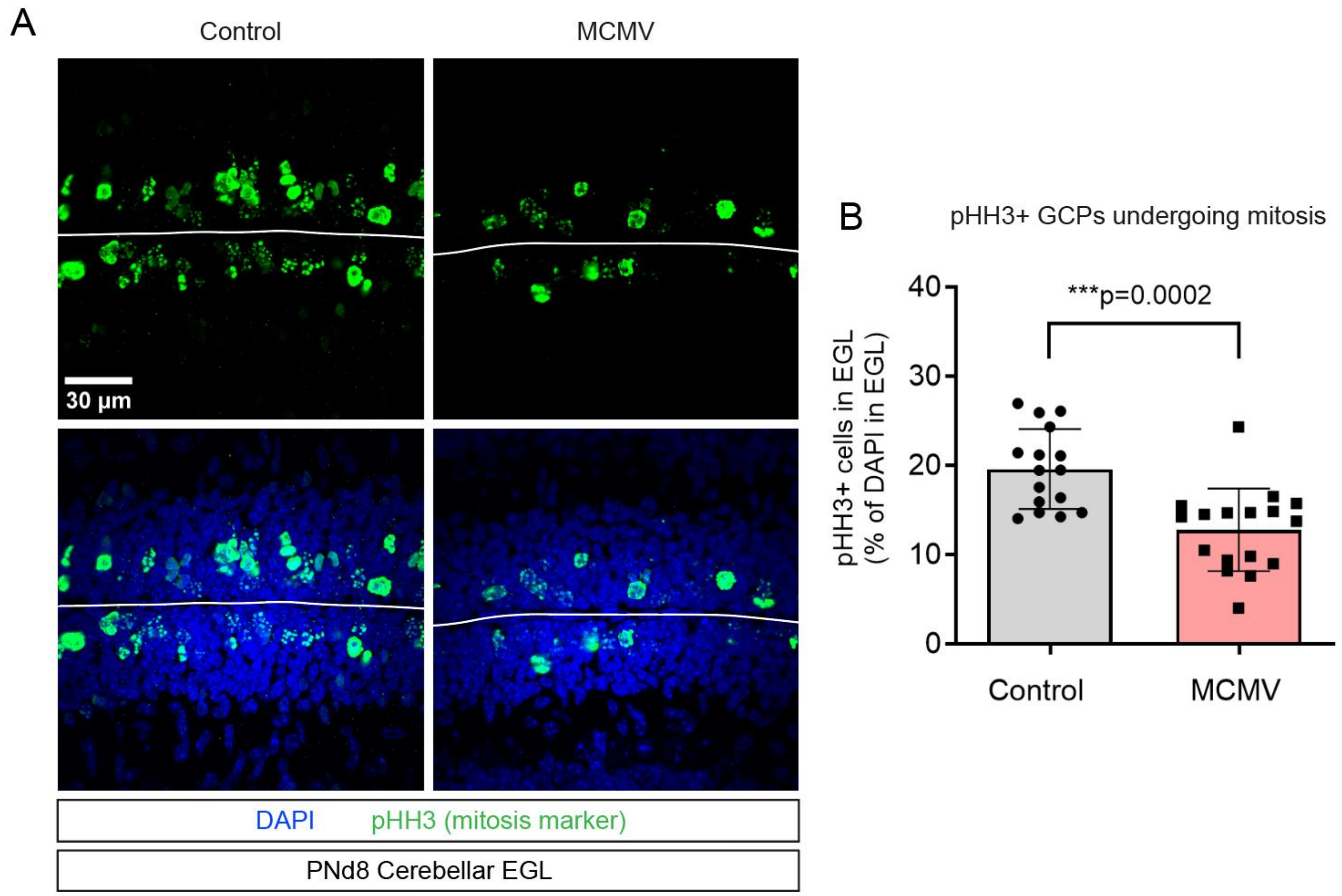

Supplemental Figure 2. Fewer GCPs were in G2/M-phase of MCMV-infected cerebella. (A) Representative images of cerebella from non-infected control and MCMV-infected mice stained for phospho-histone H3 (pHH3; green) to visualize GCPs in G2/M-phase. Scale bar: 30 µm. **(B)** GCPs in G2/M-phase were quantified in percentage of $pHH3^+$ GCPs to total number of nuclei (DAPI; blue) in the EGL ($pHH3^+$/total DAPI in the EGL). Images of cerebellar fissure are represented as two data points. Data are shown as mean ± SD, n=8-9 mice/experimental group in three different regions in the cerebellum. P-values were calculated using two-tailed t test.

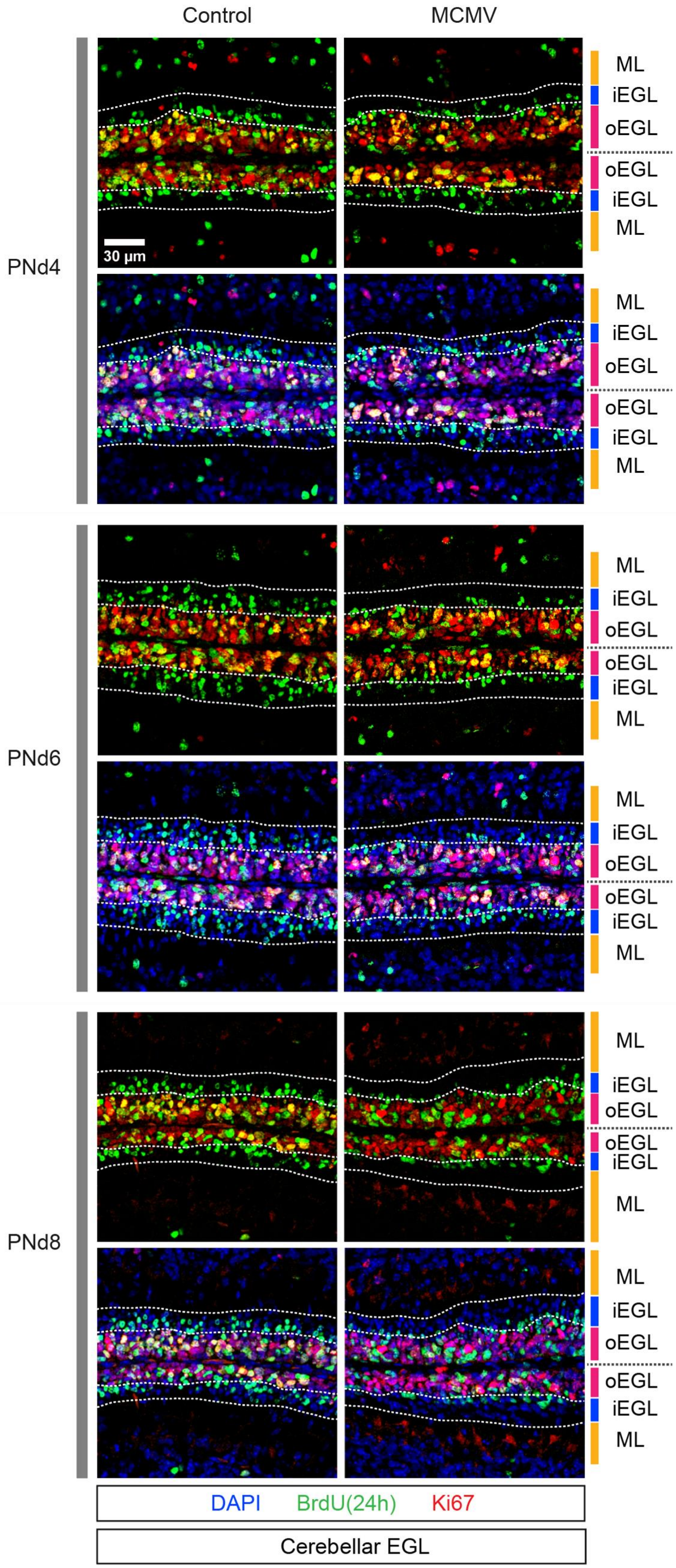

Supplemental Figure 3. Cerebellar GCP cell cycle exit is increased, and migration of GCPs from oEGL to iEGL is delayed. After BrdU-incorporation for 24 hrs at PNd4, 6, and 8, brains were harvested to assess the cell cycle exit and GCP migration. Brain sections were stained with anti-Ki67 and anti-BrdU antibodies. Representative images demonstrate that the number of cells that exited cell cycle in the EGL ($BrdU^{+}Ki67^{-}$, green) was moderately increased at PNd6 and significantly increased at PNd8 in MCMV-infected mice. In addition, the number of cells that migrated from the oEGL ($Ki67^{+}$ layer) to the iEGL (between two white dotted lines, $Ki67^{-}$ layer) was reduced in cerebella from PNd6 and PNd8 MCMV-infected mice. Please find the schematic diagram illustrating the 24-hour BrdU-incorporation protocol in Figure 3C and quantifications for cell cycle exit and cell cycle re-entry in Figure 3D-E. Scale bar: 30 μm

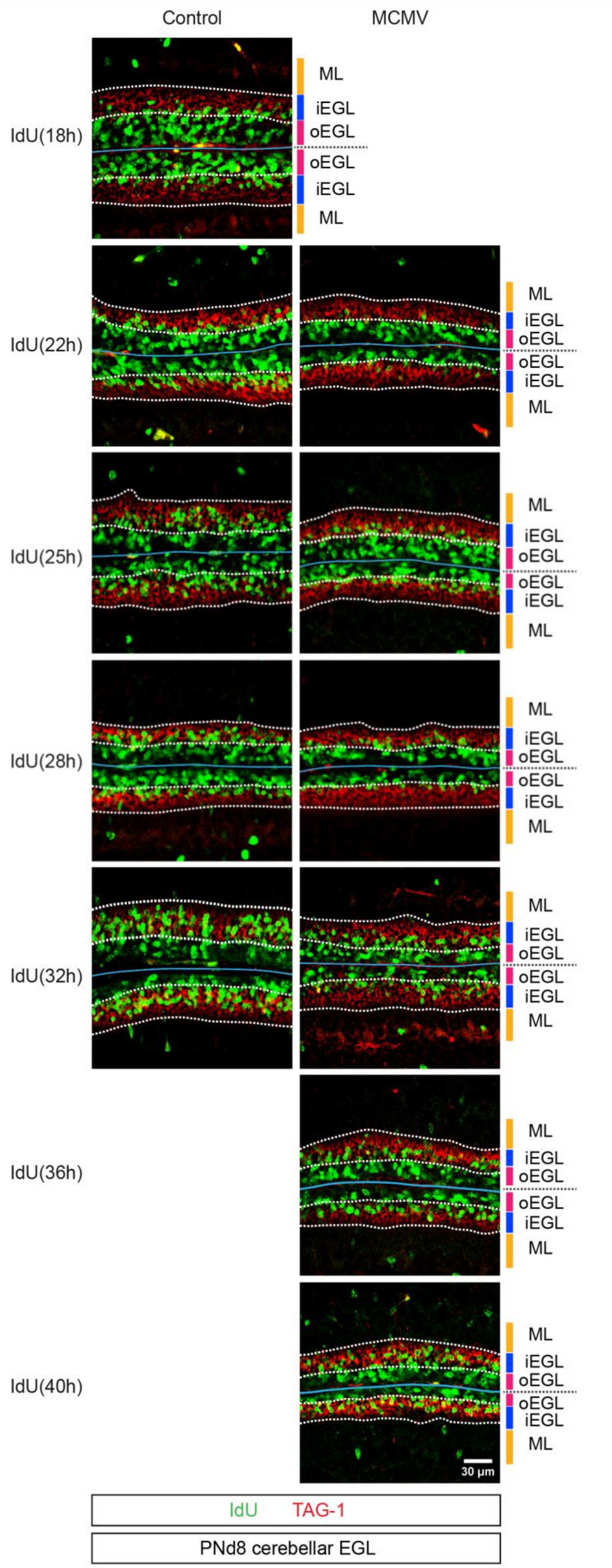

Supplemental Figure 4. Migration of GCPs from oEGL to iEGL is delayed in MCMV-infected cerebellum. Representative images of cerebella from non-infected and MCMV-infected mice stained for IdU (green) and TAG-1 (red) after treatment with IdU for 18, 22, 25, 28, 32, 36, and 40 hours to track migration of GCPs. TAG-1 is a cell adhesion molecule highly expressed in axons of immature/mature GCs and specifically stains for the iEGL. Cerebellar folia are indicated by the white solid line and the iEGL is located between the two white dotted lines (TAG-1^{+} layer). Outer EGL (oEGL, magenta label); inner EGL (iEGL, blue label); molecular layer (ML, orange label). Data are representative images of n=4-6 mice/experimental group.

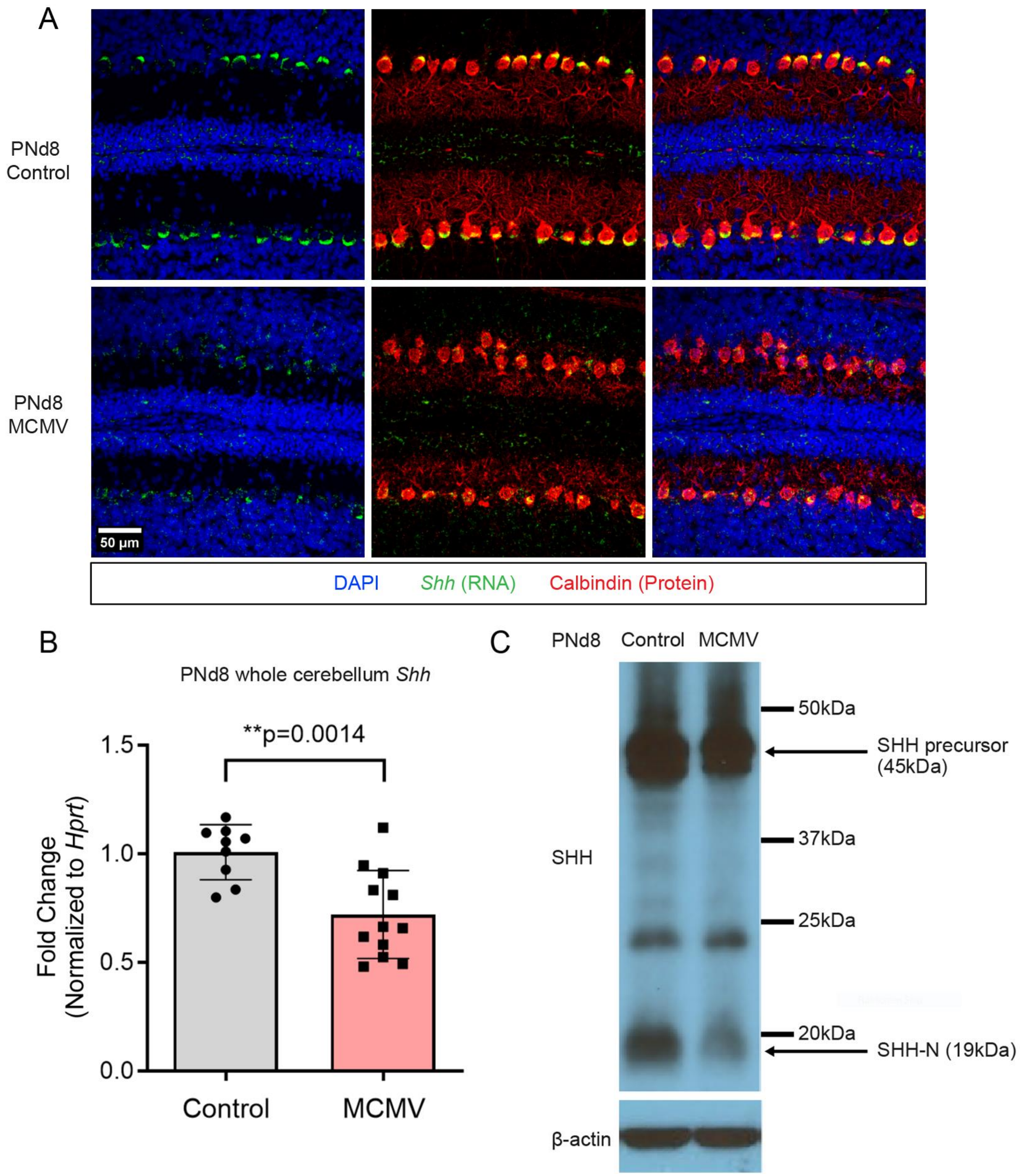

Supplemental Figure 5. Sonic hedgehog (SHH) expression is decreased in MCMV-infected cerebella. (A) RNA *in situ* hybridization on PNd8 brain sections show reduced expression of *Shh* mRNA (green) in the Purkinje cells (red) detected with antibody against Calbindin in MCMV-infected mice. Scale bar: 50 µm. **(B)** RT-PCR and **(C)** western blotting of PNd8 whole cerebella confirmed the reduction of *Shh* mRNA expression and Shh protein level in the MCMV-infected mice. Data are shown as mean ± SD, n=4-6 mice/experimental group for immunofluorescence, n=8-12 mice/experimental group for RT-PCR, and 4-5 samples (4 cerebella pooled for each sample)/experimental group were used for western blot analysis. P-values were calculated using two-tailed t-test (*p<0.05; **P<0.01 ; ****p<0.0001).

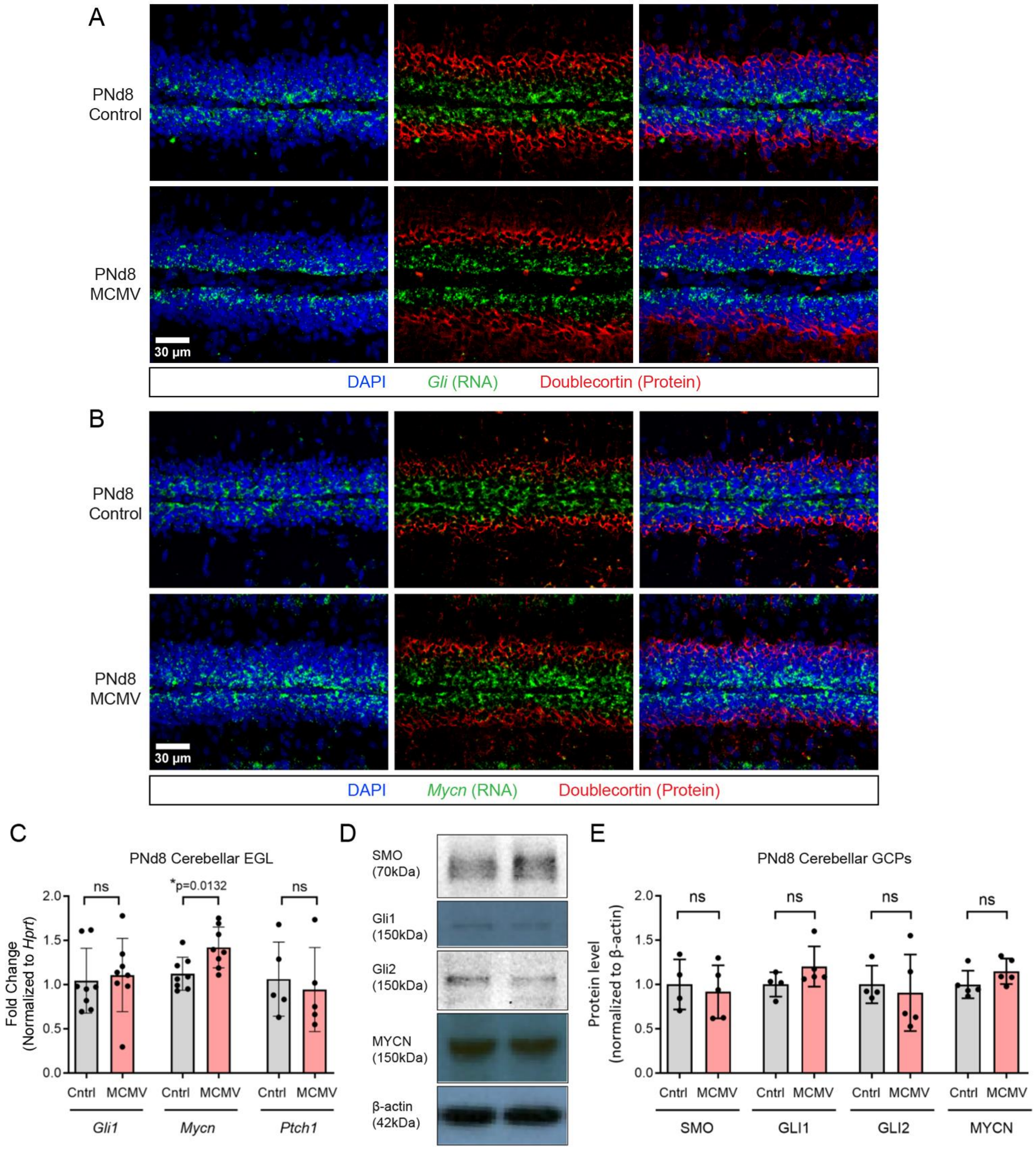

Supplemental Figure 6. SHH signaling pathways in GCPs of MCMV-infected cerebella are unaltered. (A) RNA *in situ* hybridization shows comparable expression of **(A)** *Gli1* (green) and **(B)** *Mycn* (green), downstream transcription factors in SHH pathway. PNd8 brain sections were immunostained for doublecortin (red) to label immature/mature differentiated GCs in the iEGL in the cerebellum. **(A-B)** Scale bar: 30 µm. **(C-E)** Genes and proteins downstream of SHH shows similar levels between MCMV-infected and non-infected control cerebella. **(C)** *Gli1*, *Mycn*, and *Ptch1* transcript levels were measured from RNA isolated from laser microdissected cerebellar EGL. Note elevation of *Mycn* transcript level from EGL of MCMV-infected mice compared to control mice**. (D-E)** Protein levels of SMO, GLI1, GLI2, and MYCN were measured and quantified from primary GCPs isolated from MCMV-infected and non-infected control cerebella. Data are shown as mean ± SD, n=4-6 mice/experimental group for RNA *in situ* hybridization/immunofluorescence, n=8-12 mice/experimental group for RT-PCR, and 4-5 samples (4 cerebella pooled for each sample)/experimental group were used for western blot analysis. P-values were calculated using two-tailed t-test (*p<0.05; **P<0.01; ****p<0.0001).

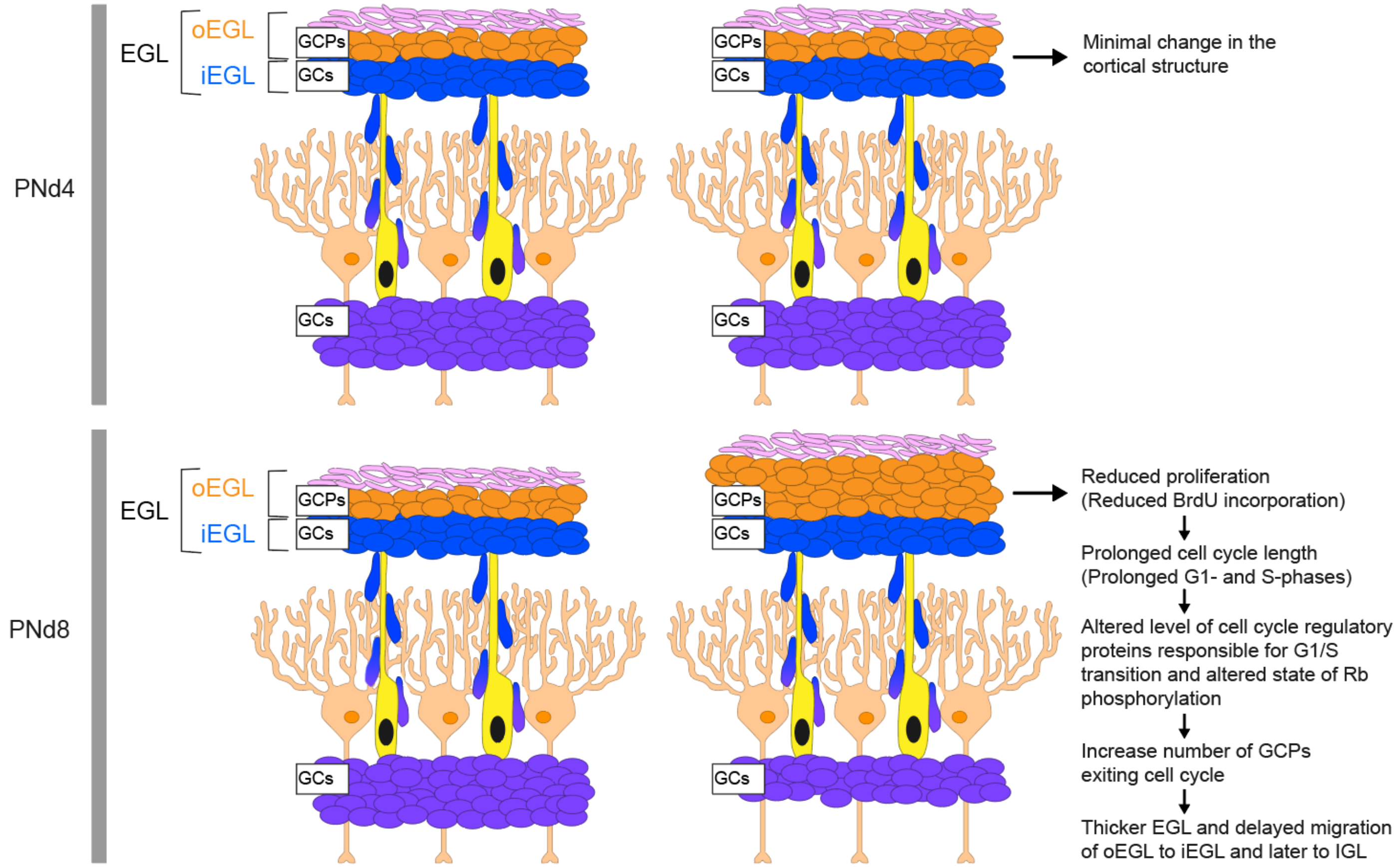

Supplemental Figure 7. Working model of the mechanisms underlying MCMV-induced disruption of cerebellar cortical structure. MCMV infection has minimal impact on cerebellar development at PNd4. However, at PNd8, MCMV infection and its consequent TNFα-regulated inflammatory responses leads to reduced GCP proliferation, accompanied by prolonged G1- and S-phases of the cell cycle, and increased number of GCPs prematurely exiting the cell cycle. This disruption in the cell cycle of GCPs results in a delayed migration of GCPs from the oEGL to the iEGL, and subsequently to the IGL, contributing to disorders involving cerebellar hypoplasia in infected animals.

Supplemental Table

Supplemental Table 1. Primary antibodies used in this study.

Antigen	Clone	Species	Isotype	Experiment	Company	Catalog #	Accession #
BrdU	BU1/75(ICR1)	Rat	IgG2a	IF	Abcam	ab6326	RRID:AB_305426
Ki-67		Rabbit	polyclonal	IF	Abcam	ab66155	RRID:AB_1140752
Doublecortin (DCX)		Rabbit	polyclonal	IF	Abcam	ab18723	RRID:AB_732011
Doublecortin (DCX)	E-6	Mouse	IgG1	IF	Santa cruz	sc-271390	RRID:AB_10610966
TAG-1	4D7	Mouse	IgM	IF	Iowa Hybridoma Bank		
IdU-FITC	B44	Mouse	IgG1	IF	BD Bioscience	347583	RRID:AB_400327
Calbindin-D28K	CB-955	Mouse	IgG1	IF	Sigma	C9848	RRID:AB_476894
MCMV IE-1	pp89	Mouse	IgG1	IF	Britt Lab generated	Croma 101	(3)
Iba1		Rabbit	polyclonal	IF	Wako	019-19741	RRID:AB_839504
phospho-histone H3 (ser10)		Rabbit	polyclonal	IF	Millipore/Upstate	05-817	RRID:AB_11215621
Smoothened (Smo)	E-5	Mouse	IgG2a	WB	Santa cruz	sc-166685	RRID:AB_2239686
Gli1		Rabbit	polyclonal	WB	Abcam	ab49314	RRID:AB_880198
Gli2	C-10	Mouse	IgG1	WB	Santa cruz	sc-271786	RRID:AB_10708124
phospho-Cyclin D1		Rabbit	polyclonal	WB	Cell signaling	2921S	RRID:AB_330139
Cyclin D1	92G2	Rabbit	polyclonal	WB	Cell signaling	2978S	RRID:AB_2259616
Cdk4	DCS-35	Mouse	IgG1	WB	Santa cruz	sc-23896	RRID:AB_627239
Cdk6	B-10	Mouse	IgG1	WB	Santa cruz	sc-7961	RRID:AB_627242
Cyclin E1	HE12	Mouse	IgG1	WB	Cell signaling	4129S	RRID:AB_2071200
Cdk2	D-12	Mouse	IgG1	WB	Santa cruz	sc-6248	RRID:AB_627238
E2F-1	KH95	Mouse	IgG2a	WB	Santa cruz	sc-251	RRID:AB_627476
phospho-Rb Ser780		Rabbit	polyclonal	WB	Cell signaling	9307	RRID:AB_330015
phospho-Rb Ser795	B-4	Mouse	IgM	WB	Santa cruz	sc-514031	
phospho-Rb Ser807/811		Rabbit	polyclonal	WB	Cell signaling	8516	RRID:AB_11178658
Rb	D20	Rabbit	polyclonal	WB	Cell signaling	9313	RRID:AB_1904119
ß-actin	C4	Mouse	IgG2b	WB	Millipore Sigma	MAB1501	RRID:AB_2223041

Supplemental References

1. Mandyam CD, Harburg GC, and Eisch AJ. Determination of key aspects of precursor cell proliferation, cell cycle length and kinetics in the adult mouse subgranular zone. *Neuroscience.* 2007;146(1):108-22.
2. Morris CA, Benson E, and White-Cooper H. Determination of gene expression patterns using in situ hybridization to Drosophila testes. *Nat Protoc.* 2009;4(12):1807-19.
3. Trgovcich J, Stimac D, Polic B, Krmpotic A, Pernjak-Pugel E, Tomac J, et al. Immune responses and cytokine induction in the development of severe hepatitis during acute infections with murine cytomegalovirus. *Archives of virology.* 2000;145(12):2601-18.
