# Supplementary material for: Cytomegalovirus infection lengthens the cell cycle of granule cell precursors during postnatal cerebellar development
Source: JCI Insight. 2024 Jun 10;9(11):e175525. doi: 10.1172/jci.insight.175525 (PMC11382886; doi:10.1172/jci.insight.175525)

Supplemental Figure 5

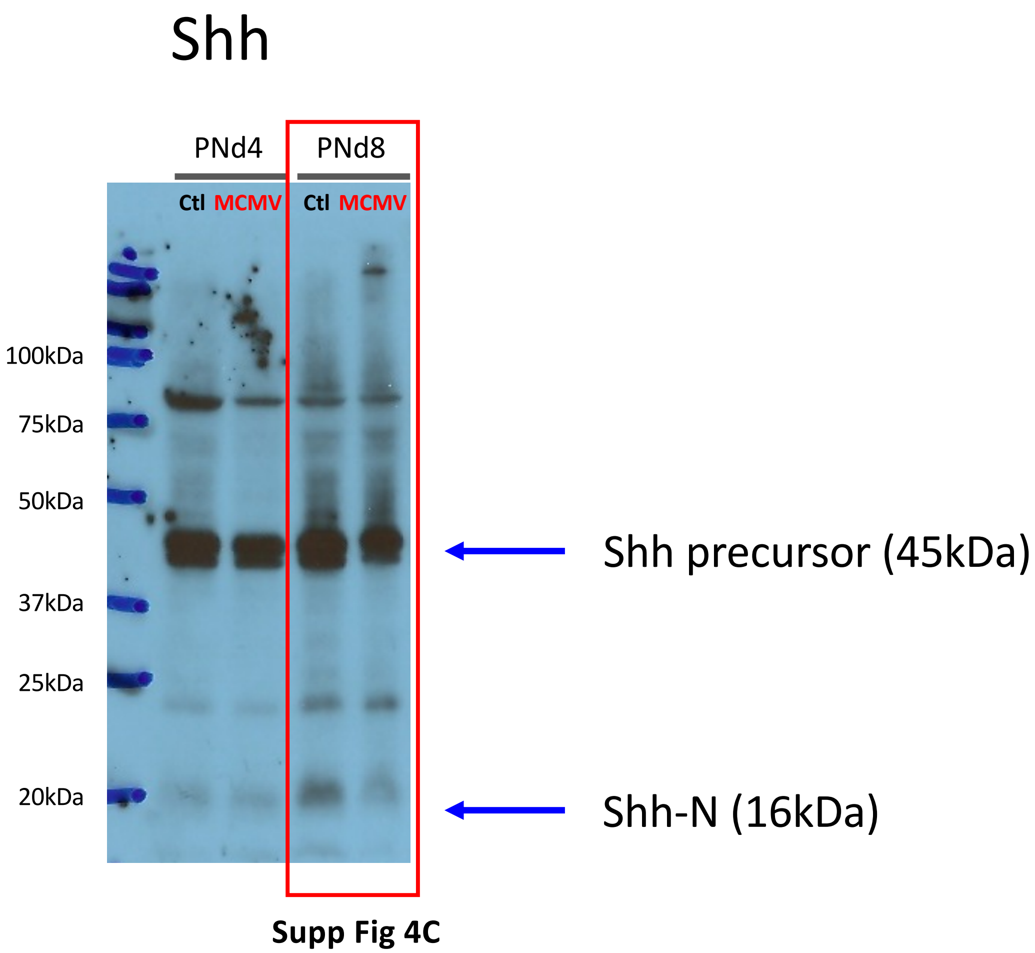

Supplemental Figure 6

Smoothened (85kDa)

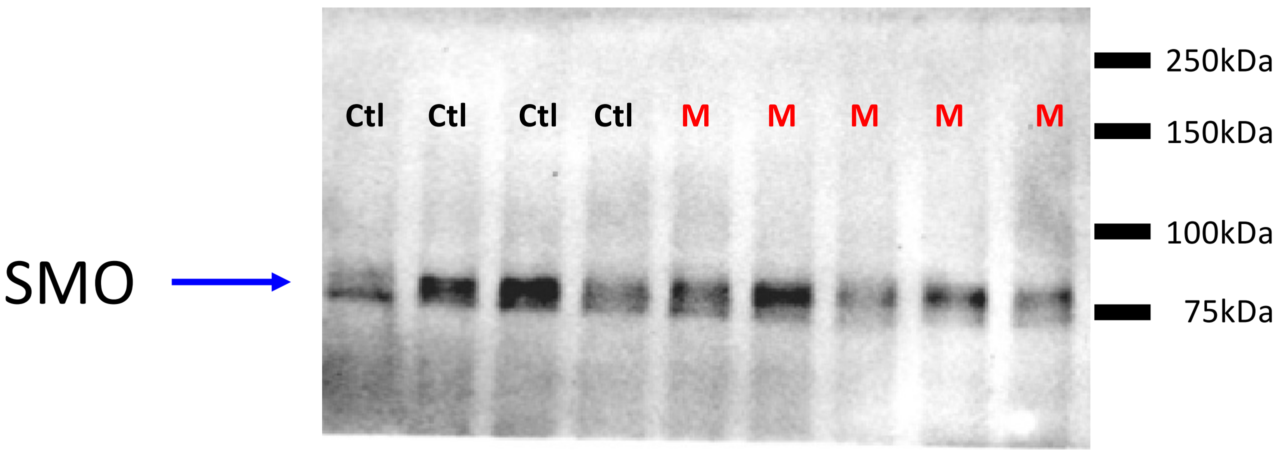

Supplemental Figure 6

Gli1 (118kDa)
Cut blot right under 75kDa

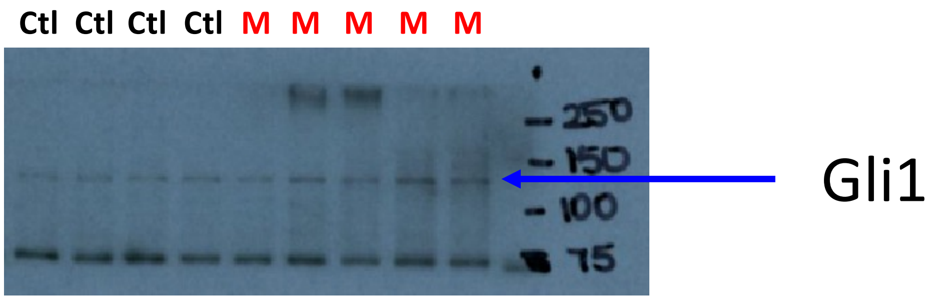

Supplemental Figure 6

Gli2

MW GLI-2 isoforms 5/a/b/g/d: 168/133/132/88/86 kDa

Cut blot right underneath 75kDa

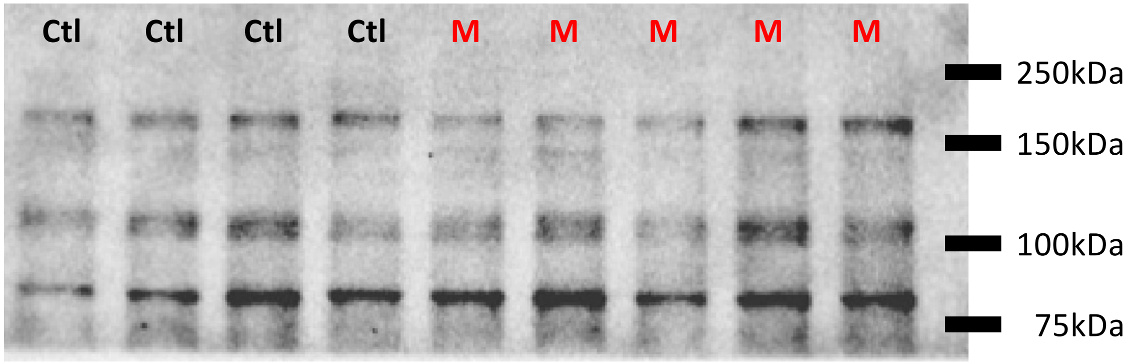

Supplemental Figure 6

MYCN (60kDa)
Cut blot right above 75kDa

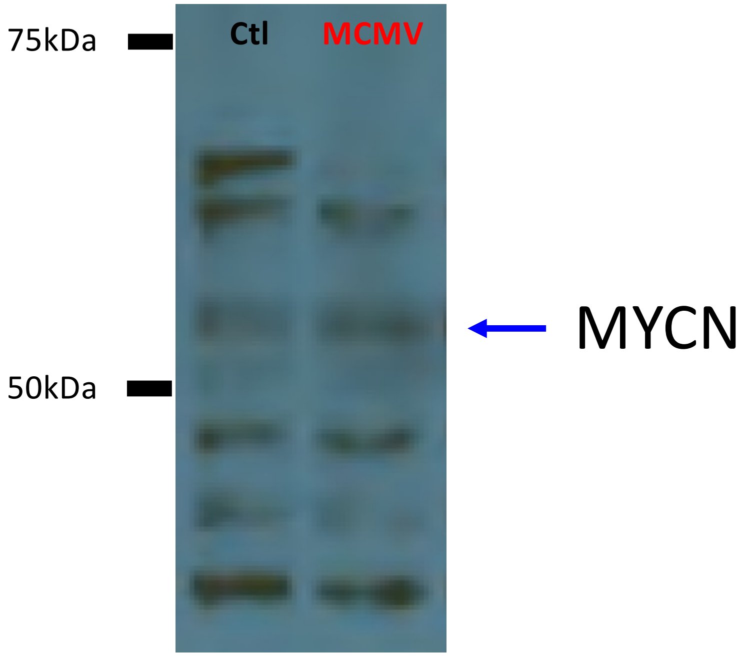

Figure 8

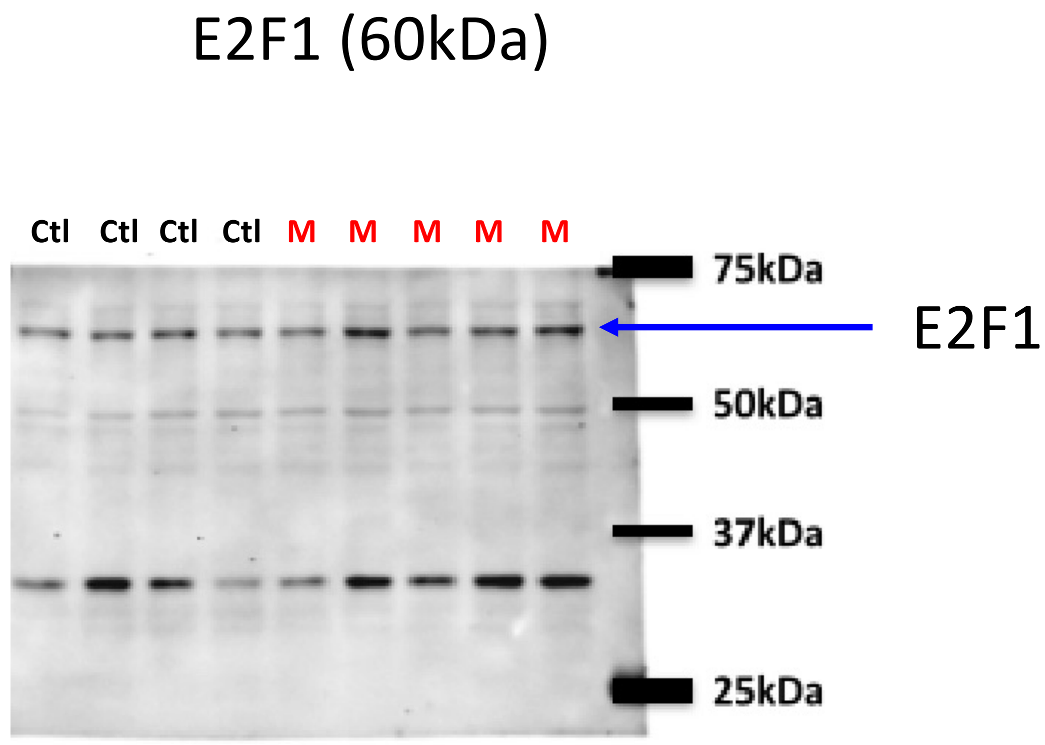

E2F1 (60kDa)
Ctl
Ctl
Ctl
Ctl
M
M
M
M
M
75kDa
E2F1
50kDa
37kDa
25kDa

Figure 8

Total Rb (110kDa)

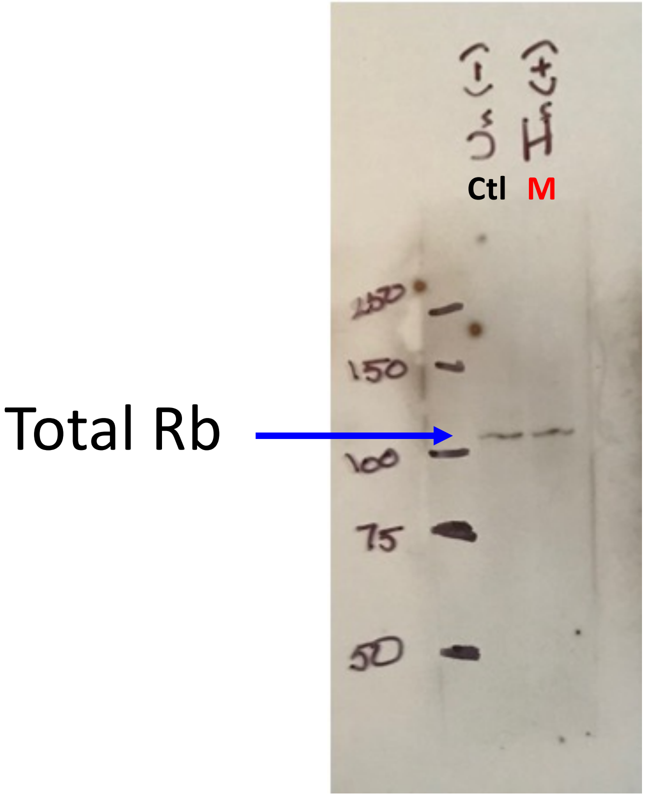

Figure 8

P-Rb Ser807/811 (110kDa)
Cut blot around 60kDa

P-Rb Ser807/811

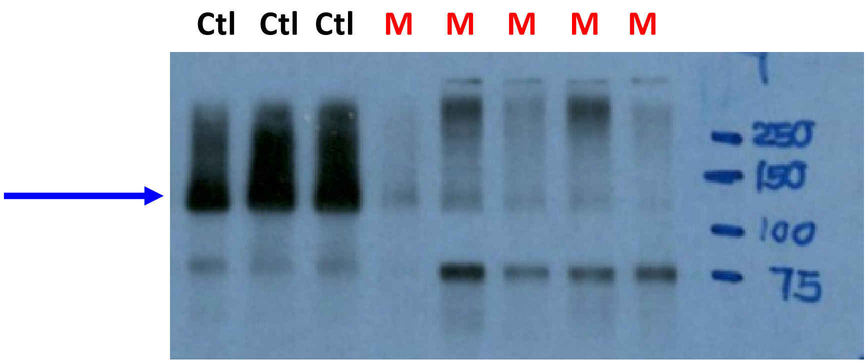

Figure 8

p-Rb S780 (top blot, 110kDa)
Cut blot between 60kDa

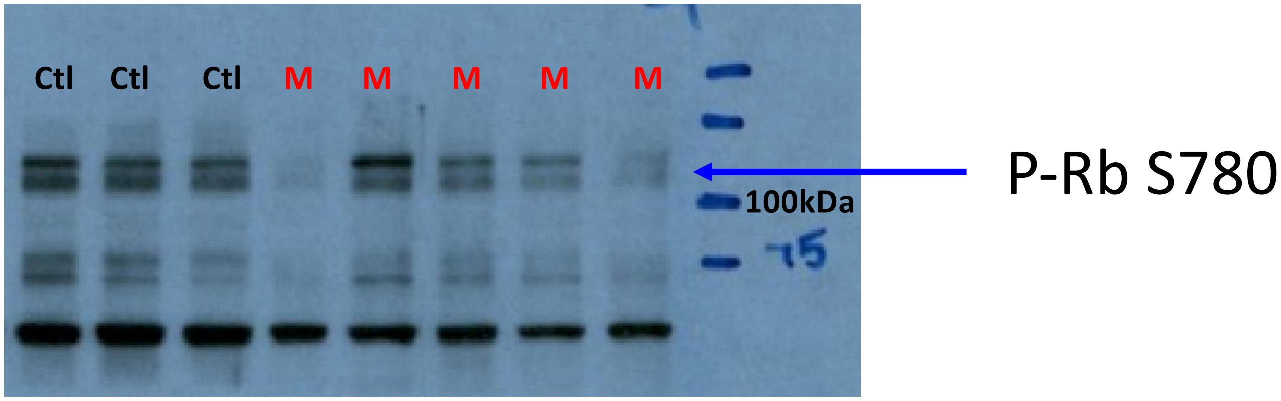

P-Rb S780

Figure 8

p-Rb S795 (top blot, 110kDa)
Cut blot between 60kDa

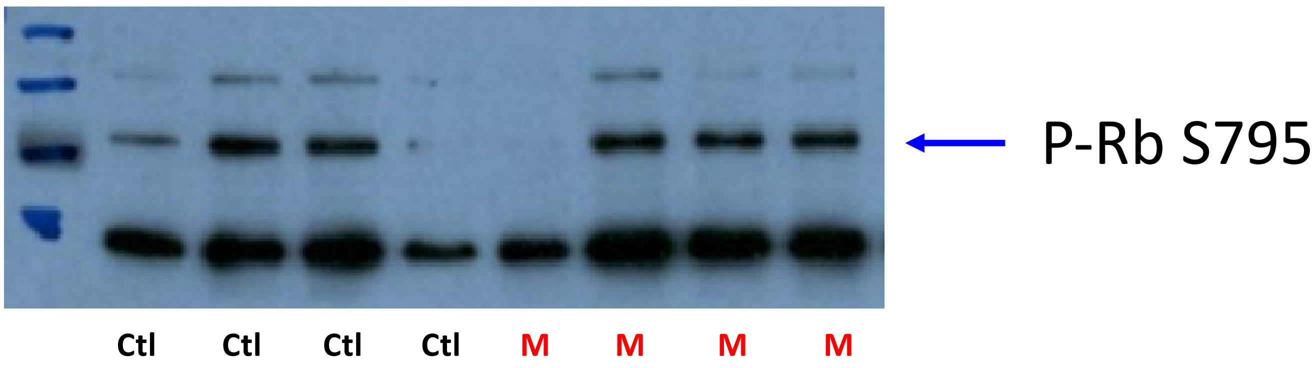

Figure 8A

β-actin (42kDa)

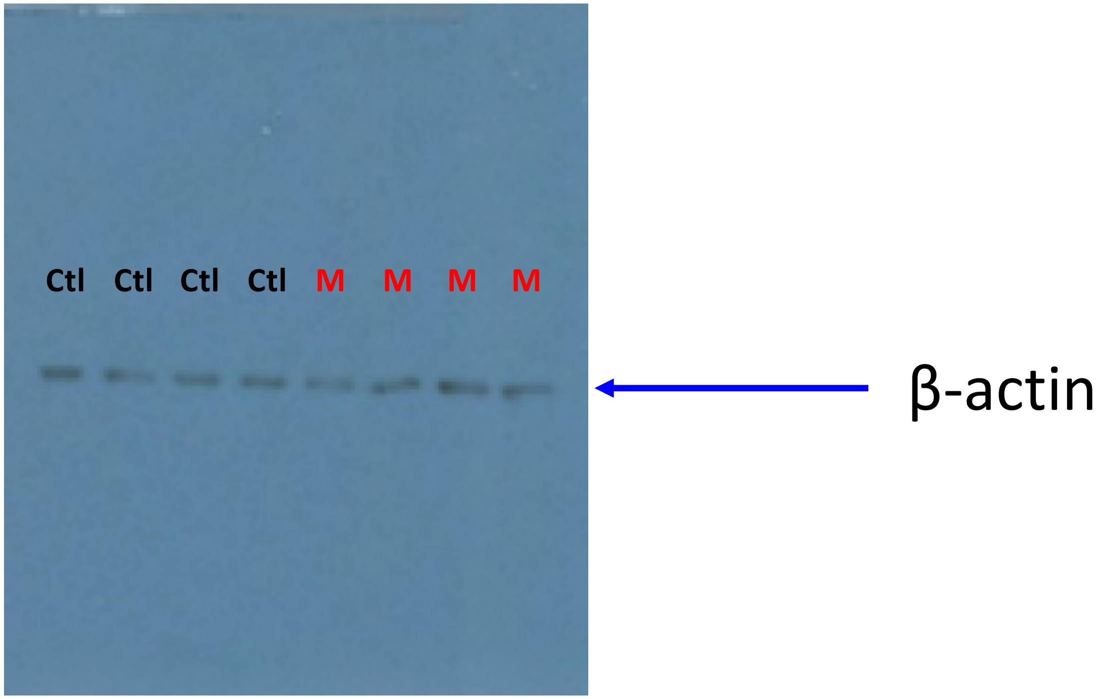

Figure 8

P-Cyclin D1 (36kDa)
Cut blot around 60kDa

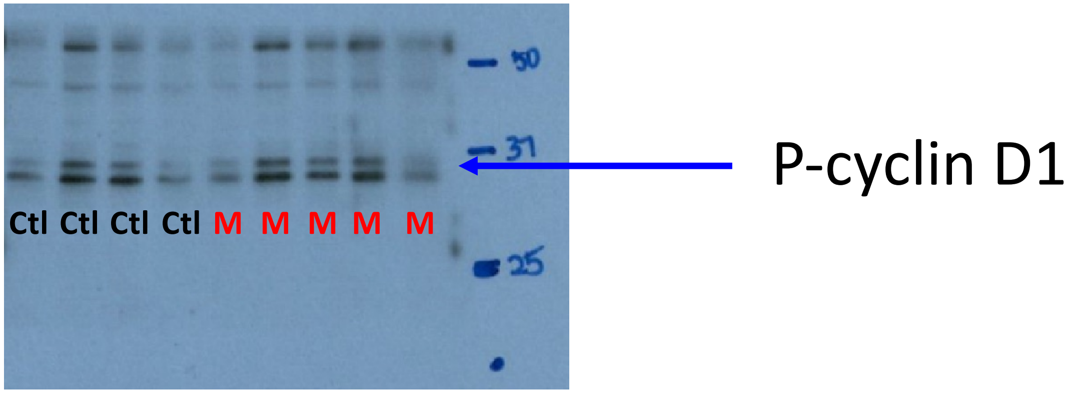

Figure 8

Cyclin D1 (36kDa)

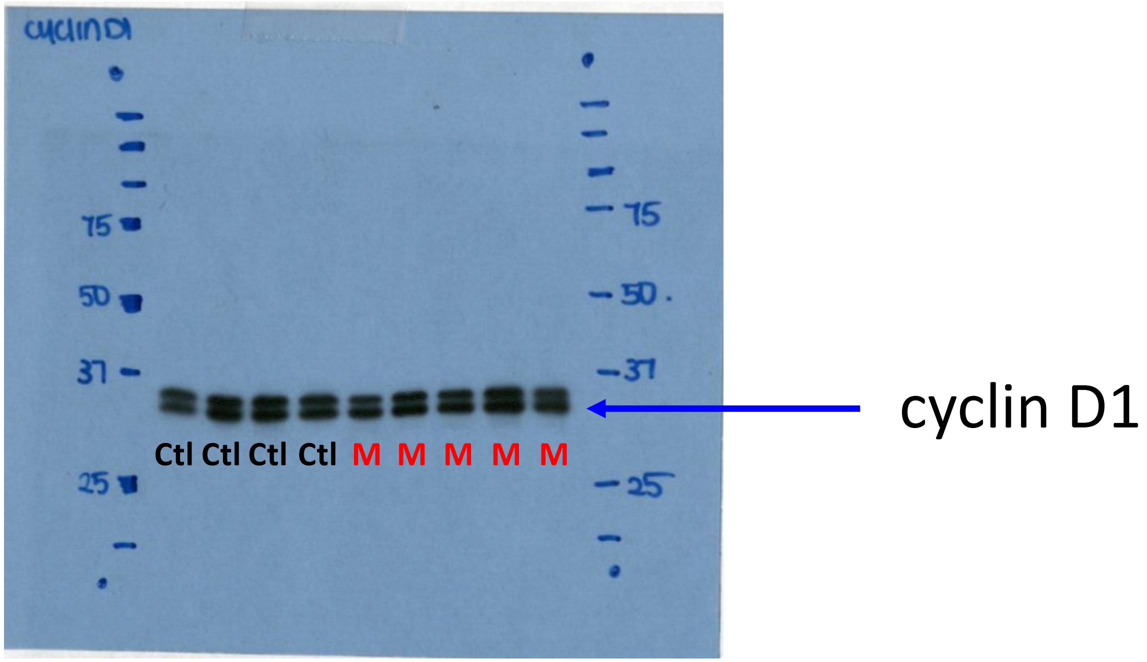

Figure 8

Cdk4 (bottom blot, 30kDa)
Cut blot around 60kDa

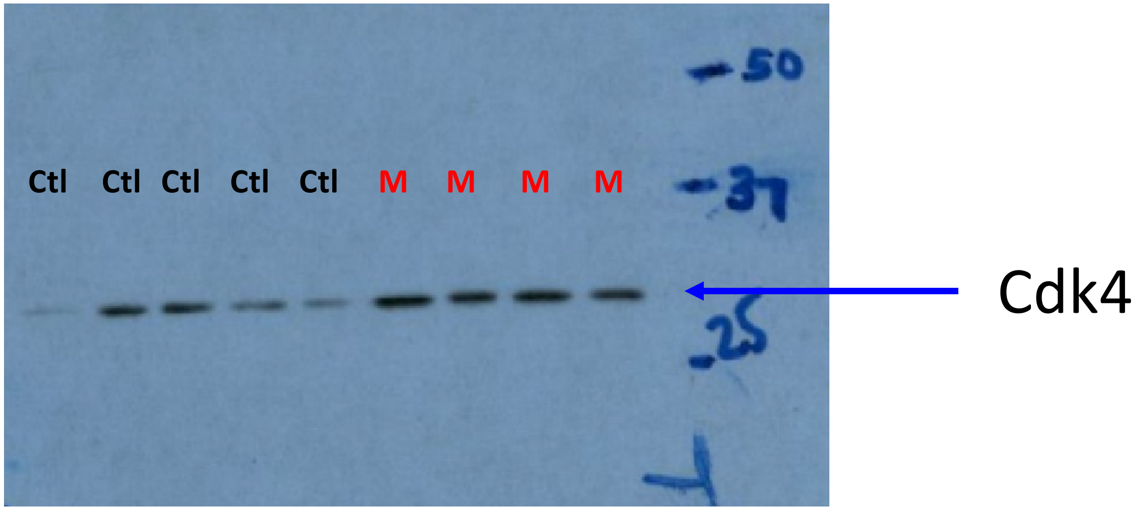

Figure 8

Cdk6 (40kDa)
Blot cut right around 50kDa

Ctl Ctl Ctl Ctl Ctl M M M M M

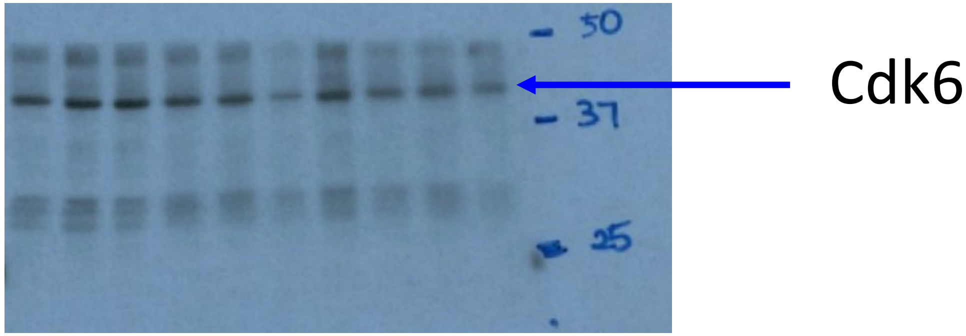

Cdk6

Figure 8

Cyclin E1 (48kDa)

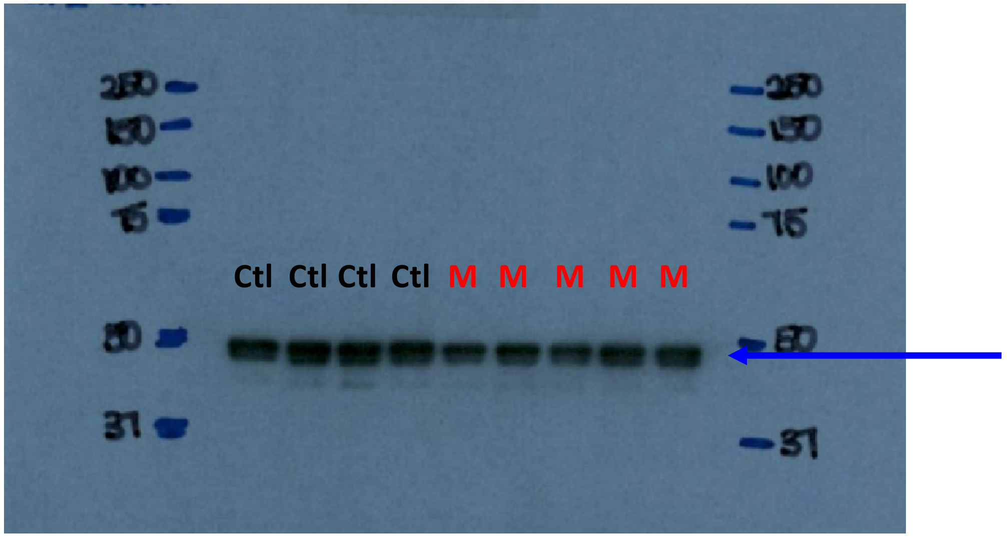

Cyclin E1

Figure 8

Cdk2 (33kDa)

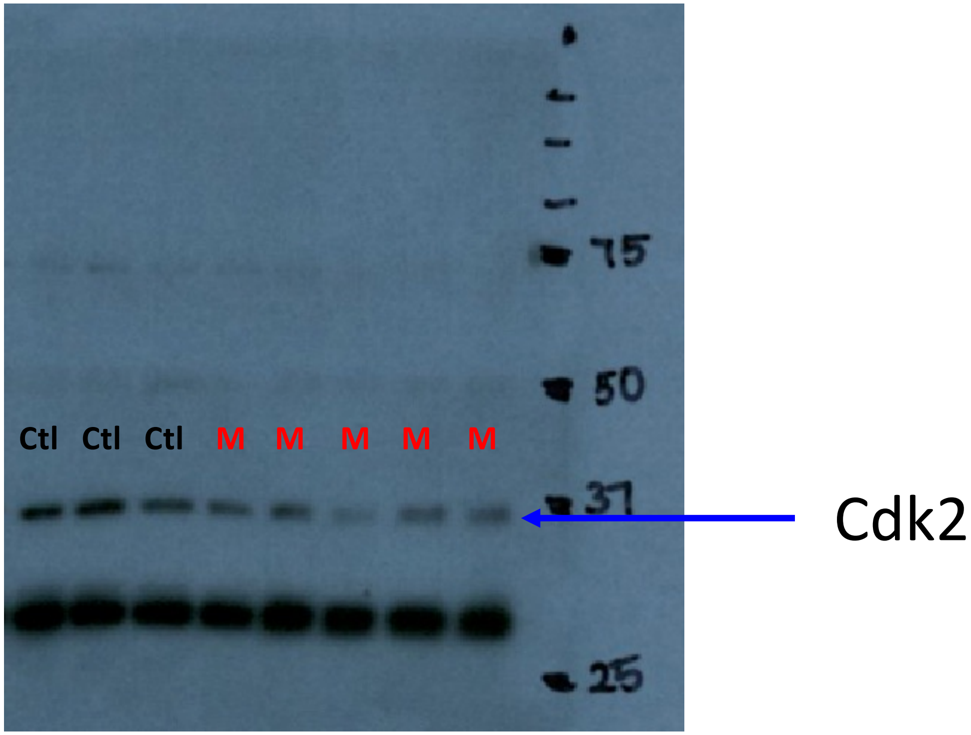

Figure 8C

β-actin (42kDa)

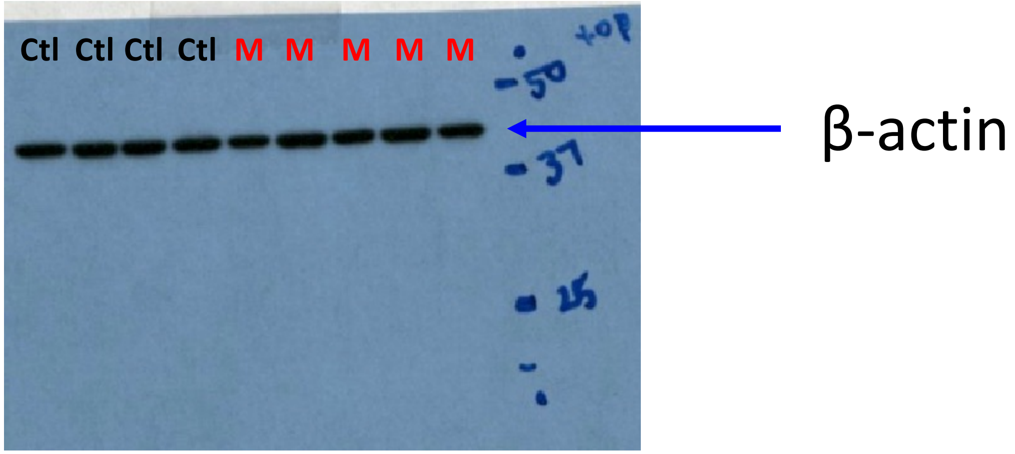

Supplement: Unedited blot and gel images [file jciinsight-9-175525-s185.pdf]
